# Cathepsin D Expression and Gemcitabine Resistance in Pancreatic Cancer

**DOI:** 10.1093/jncics/pkz060

**Published:** 2019-08-16

**Authors:** Ujjwal M Mahajan, Elisabetta Goni, Enno Langhoff, Qi Li, Eithne Costello, William Greenhalf, Stephan Kruger, Steffen Ormanns, Christopher Halloran, Paula Ganeh, Manuela Marron, Felix Lämmerhirt, Yue Zhao, Georg Beyer, Frank-Ulrich Weiss, Matthias Sendler, Christiane J Bruns, Thomas Kohlmann, Thomas Kirchner, Jens Werner, Jan G D’Haese, Michael von Bergwelt-Baildon, Volker Heinemann, John P Neoptolemos, Markus W Büchler, Claus Belka, Stefan Boeck, Markus M Lerch, Julia Mayerle

**Affiliations:** 1 Department of Medicine II, University Hospital, LMU-Munich, Munich, Germany; 2 Department of Medicine III, University Hospital, LMU-Munich, Munich, Germany; 3 Department of Radiation Oncology, University Hospital, LMU-Munich, Munich, Germany; 4 Department of Medicine A, University Medicine Greifswald, Greifswald, Germany; 5 National Institute for Health Research Liverpool Pancreas Biomedical Research Centre, University of Liverpool, UK; 6 Institute of Pathology, Faculty of Medicine, LMU Munich, Munich, Germany; 7 Leibniz Institute for Prevention Research and Epidemiology – BIPS, Bremen, Germany; 8 Department of General, Visceral, and Tumor Surgery, University Hospital Cologne, Cologne, Germany; 9 Department of Community Medicine, University Medicine Greifswald, Greifswald, Germany; 10 Department of General, Visceral, and Transplant Surgery, Ludwig-Maximilians-University Munich, Munich, Germany; 11 Department of General, Visceral and Transplantation Surgery, University of Heidelberg, Heidelberg, Germany; 12 German Cancer Consortium, German Cancer Research Center, Heidelberg, Germany

## Abstract

**Background:**

Cathepsin-D (CatD), owing to its dual role as a proteolytic enzyme and as a ligand, has been implicated in cancer progression. The role of CatD in pancreatic ductal adenocarcinoma is unknown.

**Methods:**

CatD expression quantified by immunohistochemistry of tumor-tissue microarrays of 403 resected pancreatic cancer patients from the ESPAC-Tplus trial, a translational study within the ESPAC (European Study Group for Pancreatic Cancer) trials, was dichotomously distributed to low and high H scores (cut off 22.35) for survival and multivariable analysis. The validation cohort (n = 69) was recruited based on the hazard ratio of CatD from ESPAC-Tplus. 5-fluorouracil-, and gemcitabine-resistant pancreatic cancer cell lines were employed for mechanistic experiments. All statistical tests were two-sided.

**Results:**

Median overall survival was 23.75 months and median overall survival for patients with high CatD expression was 21.09 (95% confidence interval [CI] = 17.31 to 24.80) months vs 27.20 (95% CI = 23.75 to 31.90) months for low CatD expression (χ^2^_LR, 1DF_ = 4.00; *P* = .04). Multivariable analysis revealed CatD expression as a predictive marker in gemcitabine-treated (z stat = 2.33; *P* = .02) but not in 5-fluorouracil-treated (z stat = 0.21; *P* = .82) patients. An independent validation cohort confirmed CatD as a negative predictive marker for survival (χ^2^_LR, 1DF_ = 6.80; *P* = .009) and as an independent predictive marker in gemcitabine-treated patients with a hazard ratio of 3.38 (95% CI = 1.36 to 8.38, *P* = .008). Overexpression of CatD was associated with a concomitant suppression of the acid sphingomyelinase, and silencing of CatD resulted in upregulation of acid sphingomyelinase with rescue of gemcitabine resistance.

**Conclusions:**

Adjuvant gemcitabine is less effective in pancreatic ductal adenocarcinoma with high CatD expression, and thus CatD could serve as a marker for biomarker-driven therapy.

Pancreatic ductal adenocarcinoma (PDAC) is one of the most aggressive malignancies and burdened with a 5-year survival rate of only 6% (1,2). Multiple factors are known to contribute to this dismal prognosis, with delayed diagnosis and resistance to chemotherapy or radiation therapy most prominent (3,4). The use of adjuvant chemotherapy with either 5-fluorouracil-folinic acid (5FU/FA) or gemcitabine increases estimated 5-year survival to around 17% (4,5). Use of gemcitabine did not show a survival benefit over 5FU/FA, although gemcitabine has been the treatment of choice because of its better safety profile compared to 5FU/FA (4). Even though insights into the molecular pathology of cancer can create opportunities for the development of therapies with substantial clinical benefit (6), for pancreatic cancer, such options are currently unavailable (5,7). Biomarker-driven treatment strategies are urgently needed for PDAC, but their successful development requires studies according to Reporting Recommendations for Tumor Marker Prognostic Studies (REMARK) guidelines to reduce bias (6). To identify a relevant biomarker, we used archival material from randomized, controlled trials, European Study Group for Pancreatic Cancer (ESPAC-1 [8] and 3 [4]), balanced for treatment arms, and stratified for cancer stage in resected pancreatic cancer patients.

As a candidate biomarker, we chose the lysosomal aspartic protease cathepsin-D (CatD) (9). CatD is overexpressed and hypersecreted in some epithelial cancers (10). In addition to its ubiquitous role in lysosomes, two biologic activities of the secreted precursor have been demonstrated in vitro: mitogenic activity and proteolytic activity for various substrates including proteoglycans and basement membranes in an acidic milieu. Both the growth-promoting activity and its extracellular proteolytic activity suggest that CatD may be of prognostic relevance in pancreatic cancer (9,11,12). Correlation of CatD expression with cancer progression and patient survival has been investigated in a number of solid tumors (11–14), with conflicting outcomes especially in breast cancer (15). Here, we determined whether tumor cell CatD expression predicts overall survival and treatment-related survival in patients receiving adjuvant gemcitabine or 5FU/FA in the ESPAC-Tplus trial. We validated our findings in an independent, prospectively recruited cohort and used tumor cell culture studies to identify the underlying cellular mechanisms.

## Methods

### Study Design

The translational ESPAC-Tplus studies received ethical committee approval for characterization of tumor markers for chemotherapy from the Liverpool (Adult) Research Ethics Committee (07/H1005/87). Use of good clinical practice standard operating procedures (16) ensured a full audit trail and prevented access to outcome data by pathologists and laboratory researchers. After resection for PDAC, patients in the ESPAC-3 study were randomly assigned to receive either 5FU/FA or gemcitabine. ESPAC-3 was analyzed on an intention-to-treat basis, but in the ESPAC-Tplus study, patients in the treatment arms were selected for inclusion only if treatment was actually received. This study was conducted and reported in accordance with the REMARK criteria (17–19).

For independent validation, we analyzed 69 resected patients with PDAC who were receiving adjuvant gemcitabine treatment and were recruited at the University of Munich. All the patients in the validation provided informed consent. Sample size was calculated based on the hazard ratio (HR) in the standard Cox proportional hazard model of CatD expression calculated from the multivariable analysis in the gemcitabine-treated arm (20). Details of tissue microarray manufacturing are described in Supplementary Materials and Methods (available online).

### Immunohistochemistry

Immunohistochemistry of 2 μm sections of the tissue microarrays core for CatD (goat polyclonal CatD, G20, Santacruz, 1:100 diluted in phosphate-buffered saline) was performed as described previously (21,22). The methodology adopted for scoring and H score calculations are described in Supplementary Materials and Methods (available online).

### Statistical Analysis

The first null hypothesis was that CatD levels do not predict survival in patients receiving adjuvant chemotherapy. All analyses were carried out using R (R statistical computing environment) 3.4.4 GUI 1.70 El Capitan build (7507) and R studio 1.1.442 on an intention-to-treat basis, retaining patients in their randomly assigned treatment groups and including noneligible patients. Features of statistical analyses are described in Supplementary Materials and Methods (available online). A two-sided statistical significance (*P* < .05) was used throughout.

### Cell Lines and In Vitro Experiments

The pancreatic adenocarcinoma cell lines PaTU-8988T, BxPC3, and L3.6pl were used for in vitro experiments. L3.6pl cells were received from Prof Christine J. Bruns and maintained as described previously (23). The detailed methodology for in vitro experiments is described in the Supplementary Materials and Methods (available online).

## Results

### Patients and Tissue Samples

**Figure 1.**
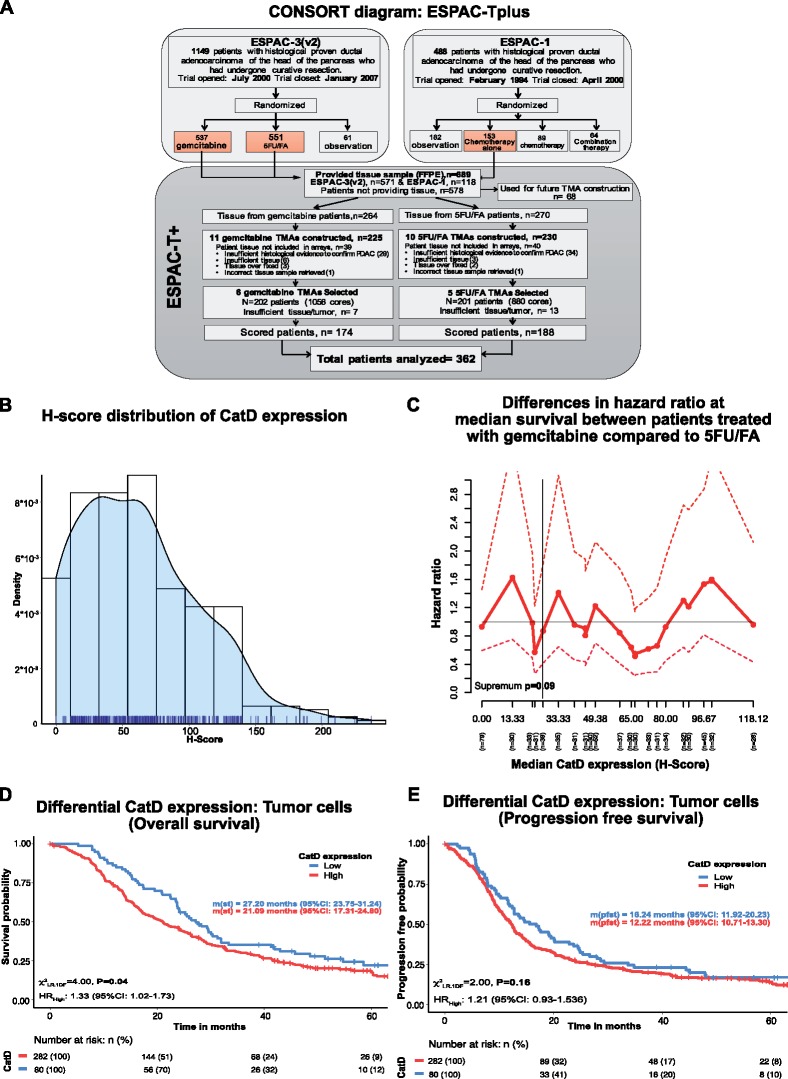
Association of CatD expression with overall survival and progression-free survival in resected PDAC patients (ESPAC cohort). **A)** CONSORT diagram. **B)** Relative frequency distribution and rug plot of CatD H-score in 362 patients. **C)** The plot shows the actual differences in risk at median survival in various CatD expression subgroups between the 5FU/FA- and gemcitabine-treatment groups (**solid line**) with a 95% CI (**dashed lines**). **Solid red horizontal line** indicates overall treatment effect, and **dotted vertical lines** indicate the cutoff for the dichotomization. *P* values are from interaction test with bootstrapping. **D)** Survival curves dichotomized by CatD expression levels (high = median H score > 22.35; low = mean H score ≤ 22.35). **E)** Progression-free survival dichotomized by CatD expression levels. All groups and the number of at-risk individuals are shown at the bottom of graph. All statistical tests were log-rank analyses using two-sided χ^2^ tests. *P* < .05 considered statistically significant. 5FU/FA = 5-fluorouracil-folinic acid; CatD = cathepsin D; CI = confidence level; CONSORT = Consolidated Standards of Reporting Trials; ESPAC = European Study Group for Pancreatic Cancer; FFPE = formalin-fixed paraffin-embedded; m(st) = median survival time; m(pfst) = median progression-free survival time; PDAC = pancreatic ductal adenocarcinoma; TMA = tissue microarray.

For the identification study, tissue microarrays of 403 patients from the ESPAC-1 and ESPAC-3 trials were selected representing 202 gemcitabine-treated and 201 5FU/FA-treated patients (Figure 1A). In the final analysis, 362 patients and 1789 tissue microarray cores were included. The demographics, surgery, and pathology reports of these patients are shown in Table 1.

**Table 1.** Demographics, surgery, and pathology features of the patients scored for CatD*

Demographics	Total No. (%) (n = 362)		Chemotherapy		
			5FU/FA (%) (n = 174)	Gemcitabine (%) (n = 188)	
Age, median (IQR), y	64 (32–83)		63 (38–83)	64 (32–82)	
Sex					
Female	153 (42.30)		75 (43.10)	78 (41.50)	
Male	209 (57.70)		99 (56.90)	110 (58.50)	
Baseline performance score					
0	121 (33.40)		61 (35.10)	60 (31.90)	
1	195 (53.90)		90 (51.70)	105 (55.90)	
2	46 (12.70)		23 (13.20)	23 (12.20)	
Diabetic, No.	348		166	182	
No	274 (78.70)		131 (78.90)	143 (78.60)	
IDDM	43 (12.40)		20 (12.00)	23 (12.60)	
NIDDM	31 (8.90)		15 (9.00)	16 (8.80)	
Smoking, No.	326		158	168	
Never	143 (43.90)		72 (45.60)	71 (42.30)	
Past	127 (39.00)		60 (38.00)	67 (39.90)	
Present	56 (17.20)		26 (16.50)	30 (17.90)	
Postoperative CA19-9, No. Median (IQR), kU/L	265		131	134	
	28 (0–27 016)		33 (0–4258)	24.5 (0–27 016)	
Surgery to random assignment, median (IQR), d	49 (4–92)		49 (4–88)	49 (7–92)	
Surgery, No.	354		170	184	
Whipple resection	185 (52.30)		92 (54.10)	93 (50.50)	
Pylorus preserving	139 (39.30)		63 (37.10)	76 (41.30)	
Distal pancreatectomy	19 (5.40)		8 (4.70)	11 (6.00)	
Total pancreatectomy	11 (3.10)		7 (4.10)	4 (2.20)	
Extent of resection, No.	346		166	180	
Standard	262 (75.70)		135 (81.30)	127 (70.60)	
Radical	47 (13.60)		20 (12.00)	27 (15.00)	
Extended radical	37 (10.70)		11 (6.60)	26 (14.40)	
Maximum tumor diameter, No. Median (IQR), mm	342		165	177	
	30 (3–350)		27 (3–350)	30 (3–105)	
Tumor grade, No.	355		171	184	
Well	27 (7.60)		12 (7.00)	15 (8.20)	
Moderate	229 (64.50)		108 (63.20)	121 (65.80)	
Poor	99 (27.90)		51 (29.80)	48 (26.10)	
Lymph node invasion					
Negative	79 (21.80)		38 (21.80)	41 (21.80)	
Positive	283 (78.20)		136 (78.20)	147 (78.20)	
Resection margin					
Negative	202 (55.80)		90 (51.70)	112 (59.60)	
Positive	160 (44.20)		84 (48.30)	76 (40.40)	
Local invasion, No.	352		170	182	
No	189 (53.70)		92 (54.10)	97 (53.30)	
Yes	163 (46.30)		78 (45.90)	85 (46.70)	
Tumor stage, No.	358		173	185	
I	23 (6.40)		12 (6.90)	11 (5.90)	
II	98 (27.40)		47 (27.20)	51 (27.60)	
III	228 (63.70)		108 (62.40)	120 (64.90)	
IV	9 (2.50)		6 (3.50)	3 (1.60)	
CatD expression					
Low	80 (22.10)		59 (34.00)	21 (11.20)	
High	282 (77.90)		115 (66.00)	167 (88.80)	

* 5FU/FA = 5-fluorouracil-folinic acid; CatD = cathepsin D; CI = confidence interval; IDDM = insulin-dependent diabetes mellitus; IQR = interquartile range; NIDDM = noninsulin-dependent diabetes mellitus.

### Cathepsin D Labeling

The CatD antibody used for this study has been characterized for CatD specificity using immunoblotting, immunofluorescence, and immunohistochemistry in human and mouse cell lines as well as human and mouse PDAC tissues (Supplementary Figure 1, A–F, available online). Pancreatic tissue from CatD knockout mice generated previously (24) served as a control. CatD showed persistent and differential expression in PDAC specimens (Supplementary Figure 1G, available online). In 362 patients (89.10%), the overall median H score was 58.45 for ductal tumor cells, ranging from 0 to 237 (Figure 1B). To exclude direct interactions between CatD and chemotherapy, we performed a subpopulation treatment effect pattern plot analysis. This showed a trend toward higher overall survival in patients with lower CatD values, especially in gemcitabine-treated patients (Supplementary Figure 2A, available online). Further, subpopulations with high CatD expression, considering 5F-treated patients as baseline, did not reveal actual differences in risk for median survival in various CatD expression subgroups between 5FU/FA- and gemcitabine-treatment groups (*P* = .09; Figure 1C). Having excluded biomarker-treatment interactions, we dichotomized the patients based on CatD expression as low and high CatD-expressing patients. Patients expressing CatD in tumor cells were subdivided using Cutoff finder (25), setting an overall median H-score of 22.35 as a threshold, and expression higher than threshold was regarded as high expression of CatD (Supplementary Figure 3, A and B, available online). In CatD-expressing patients, the event rate was 79.6%, with 228 deaths displaying high and 60 (20.4%) displaying low expression of CatD. In the gemcitabine-treatment group, we observed 152 (80.85%) deaths of which 137 (90.1%) displayed high CatD expression. In the 5FU/FA-treatment group, there were 136 (78.16%) deaths, of which 91 (66.9%) showed high CatD expression. Contingency testing of CatD levels with clinical and tumor characteristics (Supplementary Table 1, available online) did not reveal any statistically significant associations.

### Overall Survival Analysis

Median overall survival for patients with CatD expression was 23.75 (95% confidence interval [CI] = 20.63 to 26.11) months. Median overall survival of patients with low CatD expression was 27.20 (95% CI = 23.75 to 31.24) months compared to that of high CatD expression with 21.09 (95% CI = 17.31 to 24.80) months and a hazard ratio of 1.33 was calculated (95% CI = 1.02 to 1.73, χ^2^ = 4.00; *P* = .04) (Figure 1D). To assess the influence of CatD expression on treatment, we performed LIFETEST analysis (Supplementary Figure 4A, available online). Median overall survival for the gemcitabine-treated patients was 23.78 (95% CI = 19.25 to 26.34) months compared to 22.60 (95% CI = 18.43 to 27.30) months for patients treated with 5FU/FA. Median survival in patients treated with 5FU/FA with high or low CatD expression was 21.45 (95% CI = 15.90 to 27.49) and 25.23 (95% CI = 17.45 to 30.39) months, respectively (χ^2^_LR, 1DF_ = 1.25, *P* = .26) (Supplementary Figure 4B, available online). Importantly, median survival for the patients treated with gemcitabine with low CatD expression was 31.24 (95% CI = 24.11 to 51.31) months compared to 20.97 (95% CI = 16.68 to 25.13) months in those with high CatD expression, with a hazard ratio of 1.66 (95% CI = 1.06 to 2.58, χ^2^_LR, 1DF_ = 3.60, *P* = .05) (Supplementary Figure 4C, available online). Survival between the two treatment regimens did not differ with respect to CatD expression (Supplementary Figure 4, D and E, available online).

Median progression-free survival for patients with CatD expression was 12.74  (95% CI = 11.63 to 14.19) months. Median progression-free survival of patients with low CatD expression was 16.24 (95% CI = 11.92 to 20.23) months compared to that of high CatD expression with 12.22 (95% CI = 10.71 to 13.30) months, with a hazard ratio of 1.21 (95% CI = 0.93 to 1.53, χ^2^ = 2.00, *P* = .16) (Figure 1E). Progression-free survival between the two treatment arms did not differ by differential CatD expression (Supplementary Figure 5, available online). Pairwise comparisons of all variations of CatD level and treatment arms did not show any association with overall survival as well as progression-free survival (Supplementary Table 1, available online).

In performing univariate analysis with overall survival as the endpoint, we observed significant associations with independent variables such as local invasion, lymph node invasion, postoperative CA19.9, resection margin, and tumor stage. CatD expression showed a statistically significant correlation with patients’ survival (Table 2). Progression-free survival did not show any significant association (Supplementary Table 3, available online).

**Table 2.** Univariate analysis of overall survival factors*

Characteristics	All patients (n = 362)			5-fluorouracil-folinic acid (n = 174)			Gemcitabine (n = 188)		
	HR (95% CI)	χ^2^	*P*	HR (95% CI)	χ^2^	*P*	HR (95% CI)	χ^2^	*P*
Age	0.99 (0.98 to 1.00)	1.43	.23	0.98 (0.97 to 1.00)	1.33	.25	0.99 (0.97 to 1.01)	0.25	. 62
Sex , No.	362			174			188		
Female	1 (Referent)			1 (Referent)			1 (Referent)		
Male	0.91 (0.72 to 1.14)	0.68	.41	0.89 (0.63 to 1.25	0.42	.51	0.92 (0.67 to 1.27)	0.23	.63
Smoking, No.	326			158			168		
Never	1 (Referent)			1 (Referent)			1 (Referent)		
Past	1.15 (0.88 to 1.51)	1.11	.29	1.07 (0.73 to 1.59)	0.32	.70	1.23 (0.85 to 1.80)	3.89	.26
Present	1.34 (0.96 to 1.88)	2.00	.09	1.14 (0.70 to 1.87)	2.00	.59	1.57 (0.99 to 2.50)	2.00	.05
Lymph node invasion, No.	362			174			188		
Negative	1 (Referent)			1 (Referent)			1 (Referent)		
Positive	2.00 (1.47 to 2.73)	19.42	<.001	2.35 (1.48 to 3.72)	13.26	<.001	1.70 (1.12 to 2.58)	6.21	.01
Resection margin, No.	362			174			188		
Negative	1 (Referent)			1 (Referent)			1 (Referent)		
Positive	1.56 (1.24 to 1.97)	14.01	<.001	1.74 (1.24 to 2.45)	10.24	.001	1.40 (1.01 to 1.93)	4.18	.04
Local invasion	352			170			182		
No	1 (Referent)			1 (Referent)			1 (Referent)		
Yes	1.34 (1.06 to 1.69)	5.96	.01	1.39 (0.99 to 1.95)	3.66	.05	1.29 (0.94 to 1.79)	2.49	.11
Tumor stage, No.	358			173			185		
I	1 (Referent)			1 (Referent)			1 (Referent)		
II	1.67 (0.91 to 3.08)	12.65	.09	2.21 (0.87 to 5.64)	7.39	.09	1.25 (0.56 to 2.80)	5.81	.59
III	2.29 (1.27 to 4.10)	3.00	.01	3.00 (1.22 to 7.43)	3.00	.02	1.71 (0.80 to 3.69)	3.00	.17
IV	1.29 (0.48 to 3.44)	0.005	.61	2.28 (0.66 to 7.90)	1.19	.06	0.42 (0.05 to 3.47)	0.12	.43
Postoperative CA19-9, No.	265			131			134		
	1.00 (1.00 to 1.00)	22.21	<.001	1.00 (1.00 to 1.00)	14.67	<.001	1.00 (1.00 to 1.00)	14.29	<.001
Maximum tumor size, No.	342			165			177		
	1.00 (0.99 to 1.01)	2.66	.10	1.01 (0.99 to 1.01)	1.69	.19	1.00 (0.99 to 1.01)	0.64	.42
Differentiation status, No.	355			171			184		
Well	1 (Referent)			1 (Referent)			1 (Referent)		
Moderate	1.05 (0.67 to 1.65)	2.98	.83	1.03 (0.51 to 2.05)	0.08	.93	1.06 (0.58 to 1.93)	5.22	.85
Poor	1.31 (0.81 to 2.12)	2.00	.27	1.08 (0.52 to 2.24)	2.00	.83	1.60 (0.84 to 3.06)	2.00	.15
CatD expression, No.	362			174			188		
Low	1 (Referent)			1 (Referent)			1 (Referent)		
High	1.34 (1.00 to 1.77)	3.98	.04	1.22 (0.86 to 1.75)	1.25	.26	1.66 (0.97 to 2.84)	3.51	.05

* CatD = cathepsin D; CI = confidence interval; HR = hazard ratio.

### Multivariable Analysis

**Figure 2.**
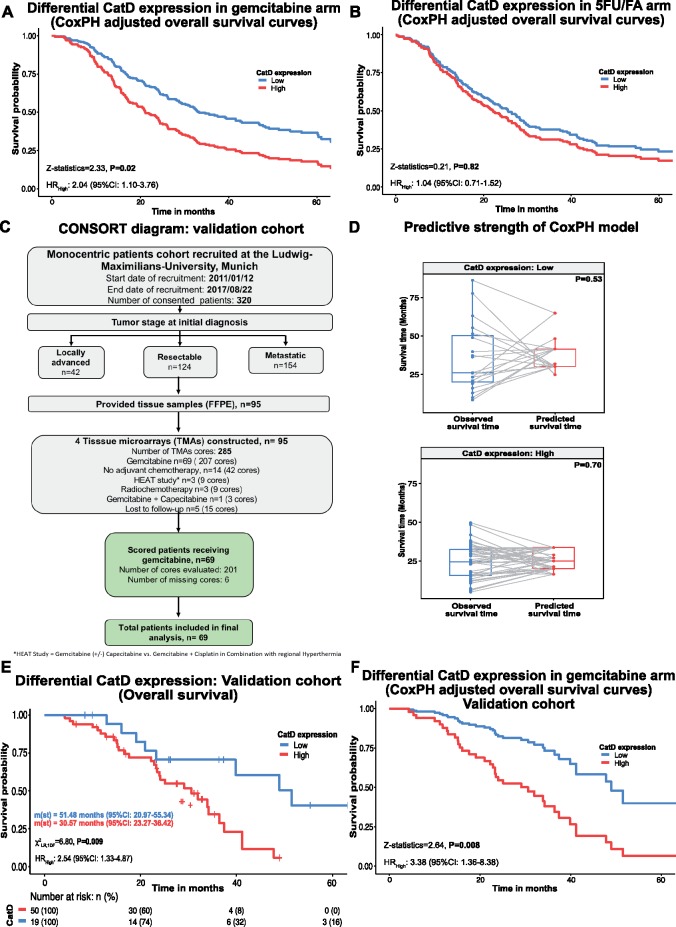
Association of CatD expression with overall survival in resected PDAC patients in multivariable analysis and in an independent validation cohort. **A)** Multivariable Cox proportional hazard regression analysis for overall survival stratified for gemcitabine treatment with CatD expression as one of the covariates along with resection margin, smoking status, lymph node invasion, and local invasion status as other independent covariates. **B)** Multivariable Cox proportional hazard regression analysis for overall survival stratified for 5FU/FA treatment arm with CatD expression as one of the covariates along with sex, smoking status, lymph node invasion, and local invasion status as other independent covariates. **C)** CONSORT diagram of an independent validation cohort. **D)** Box and whisker plots predicting observed survival time (**blue**) and predicted survival time (**red**) from the Cox proportional hazard model of ESPAC-Tplus cohort for the validation cohort stratified for low and high CatD expression. **Connecting lines** depict the connection of the event in observed survival time and predicted survival time. **E)** Survival curves split by CatD expression levels (high = median H score > 22.35; low = mean H score ≤ 22.35) in the validation cohort (univariate analysis). **F)** Multivariable Cox proportional hazard regression analysis for overall survival stratified for validation cohort with CatD expression as one of the covariates along with sex, lymph node invasion, and local invasion status as other independent covariates. All statistical tests were log-rank analyses using two-sided χ^2^ tests. *P* < .05 considered statistically significant. 5FU/FA = 5-fluorouracil/folinic acid; CatD = cathepsin D; CI = confidence interval; CONSORT = Consolidated Standards of Reporting Trials; ESPAC = European Study Group for Pancreatic Cancer; FOLFIRINOX = folic acid, 5-fluorouracil, irinotecan, oxaliplatin; FFPE = formalin-fixed paraffin-embedded; HR = hazard ratio; m(st) = median survival time in months; PDAC = pancreatic ductal carcinoma; PH = proportional hazard; RCT = radiochemotherapy; TMA = tissue microarray.

A model for multivariable analysis based on 318 patients (255 deaths) identified lymph node invasion, local invasion, resection margin, smoking status, and tumor cell CatD expression as independent survival factors. Cox proportional hazard model for overall survival revealed statistical significance for CatD expression in the gemcitabine-treatment arm, with a hazard ratio of 2.04 (95% CI = 1.10 to 3.76, z statistic = 2.33; *P* = .02, Figure 2A) but not in patients treated with 5FU/FA, with a hazard ratio of 1.04 (95% CI = 0.71 to 1.52, z statistic = 0.21; *P* = .82; Figure 2B, Table 3). Comparison of this Cox proportional hazard model (C-statistics = 0.62 ± 0.02) with a model leaving out CatD expression (C statistics = 0.59 ± 0.02; Supplementary Table 4, available online) in gemcitabine-treated patients showed statistically significant improvement in the stated model after the addition of CatD expression (*P* = .01). Multivariable analysis with progression-free survival factors mirrored the same trends (Supplementary Figure 4C and Supplementary Table 5, available online).

**Table 3.** Multivariable analysis of CatD with overall survival factors*

Covariates		ESPAC-Tplus cohort								Validation cohort			
		5FU/FA (n = 158)				Gemcitabine (n = 167)				Gemcitabine (n = 63)			
		HR (95% CI)		z stat	*P*	HR (95% CI)		z stat	*P*	HR (95% CI)		z stat	*P*
Resection margin													
	Negative	1.00 (Referent)				1.00 (Referent)				1.00 (Referent)			
	Positive	1.62 (1.13 to 2.33)		2.65	.007	1.27 (0.89 to 1.80)		1.36	.17	0.98 (0.39 to 2.42)		–0.03	.97
Smoking status													
	Never	1.00 (Referent)				1.00 (Referent)				—			
	Past	0.87 (0.58 to 1.29)		–0.66	.50	1.24 (0.86 to 1.85)		1.14	.22	—		—	—
	Present	0.96 (0.58 to 1.59)		–0.12	.89	1.66 (1.05 to 2.72)		2.11	.03	—		—	—
Lymph node status													
	Negative	1.00 (Referent)				1.00 (Referent)				1.00 (Referent)			
	Positive	2.21 (1.33 to 3.65)		3.08	.002	1.63 (1.04 to 2.56)		2.14	.03	1.28 (0.58 to 2.82)		0.63	.52
Local invasion													
	Negative	1.00 (Referent)				1.00 (Referent)				1.00 (Referent)			
	Positive	1.41 (0.98 to 2.02)		1.89	.05	1.49 (1.04 to 2.13)		2.21	.02	1.41 (0.69 to 2.89)		1.02	.34
CatD expression													
	Low	1.00 (Referent)				1.00 (Referent)				1.00 (Referent)			
	High	1.04 (0.71 to 1.52)		0.21	.82	2.04 (1.10 to 3.76)		2.33	.02	3.38 (1.36 to 8.38)		2.60	.008
C statistic		0.62 ± 0.02				0.62 ± 0.02				0.64 ± 0.05			
AIC		1089.96				1198.06				242.42			
Likelihood ratio test		25.79 (*P* < .001)				21.40 (*P* = .002)				9.14 (*P* = .06)			
Comparison with model leaving the CatD expression		χ^2^ = 0.04, P = .82				χ^2^ = 6.16, P = .01				χ^2^ = 8.28, P = .003			

* AIC = Akaike information criteria; CatD = cathepsin D; CI = confidence interval; ESPAC = European Study Group for Pancreatic Cancer; HR = hazard ratio.

Because the multivariable analysis considers only complete cases, we processed our data, barring postoperative CA 19-9 and excluding all units for which any of the inputs were missing. Demographics of complete cases are listed in Supplementary Table 6 (available online). Median overall survival for patients with CatD expression was 24.11 (95% CI = 20.63.0 to 27.30) months. Median overall survival of patients with low CatD expression was 29.60 (95% CI = 23.58 to 41.85) months compared to that of high CatD expression with 21.45 (95% CI = 17.80 to 25.89) months, and a hazard ratio of 1.44 was calculated (95% CI = 0.98 to 1.44, χ^2^= 4.80; *P* = .02; Supplementary Table 7, available online). Cox proportional hazard model for overall survival revealed statistical significance for CatD expression in the gemcitabine-treated complete cases with a hazard ratio of 2.24 (95% CI = 1.14 to 4.40, z statistic = 2.35; *P* = .01, Supplementary Figure 8A, available online) but not in 5FU/FA-treated patients, with a hazard ratio of 0.92 (95% CI = 0.61 to 1.40, z statistic = −0.34; *P* = .72, Supplementary Figure 8B and Supplementary Table 7, available online). Although exclusion of missing data led to loss of power in the multivariable analysis (n = 325 patients, HR for high CatD = 2.04), we did not observe a significant change in hazard ratio comparing results from the multivariable analysis of complete cases (n = 287 patients, HR for high CatD = 2.24).

### Independent Validation

The prespecified hypothesis for the independent validation cohort was based on the Cox proportional hazard model, with a hazard ratio of 2.04 in the gemcitabine-treated arm from the identification study. We calculated a sample size of 69 patients aiming for a β-error of 0.8 and an α-error of less than 0.05 and recruited 69 patients (207 cores) from PDAC patients resected in the Department of Surgery at the University of Munich and receiving adjuvant gemcitabine treatment. In total, we analyzed 201 cores (97.1%) corresponding to 69 patients (Figure 2C). The demographics of patients are shown in Supplementary Table 8 (available online). We observed 38 (76.0%) deaths, with 30 (78.9%) deaths showing high and eight (21.0%) showing low expression of CatD in the gemcitabine-treatment group. We did not observe any statistically significant associations on contingency testing of CatD levels with clinical and tumor characteristics (Supplementary Table 9, available online).

We were able to compare the ability to predict survival time of the Cox proportional hazard model excluding smoking status in the validation cohort. We did not observe any statistical difference between predicted and observed survival time, depicting robustness of the model (Figure 2D). Median overall survival of patients was 33.89 (95% CI = 23.96 to 41.16) months. The median survival for patients with low CatD expression in the validation cohort was 51.48 (95% CI = 20.97 to 55.34) months vs 30.57 (95% CI = 23.27 to 36.42) months for high CatD expression, (χ2_LR,__1DF_= 6.80; *P* = .009; Figure 2E). The difference between overall survival in the identification and validation cohort can be explained by several factors. First, excellent surgical outcomes in a high-volume tertiary-care center per se influence overall survival. Secondly, the high number of patients completing their course of adjuvant chemotherapy without dose reduction or chemotherapy breaks affects overall survival. Thirdly, careful, structured follow-up and early palliative treatment in recurrence often followed by second- or third-line palliative systemic therapy in the setting of clinical trials influenced outcome. All these factors have individually contributed to improved overall survival. We did not detect a statistically significant association between low and high CatD expression and progression-free survival, with χ^2^_LR, 1DF_ = 0.20; *P* = .61; (Supplementary Figure 9A, available online). In performing univariate analysis using overall survival as the endpoint, we observed, as expected, statistically significant associations with independent variables such as tumor grading, tumor stage, vascular invasion, perineural invasion, and CatD expression. Univariate analysis with progression-free survival as the endpoint did not show a statistically significant association (Supplementary Table 10, available online).

Multivariable analysis with sex, lymph node status, local invasion status, and CatD expression revealed CatD expression as an independent predictive marker in gemcitabine-treated patients, with a hazard ratio of 3.38 (95% CI = 1.36 to 8.38; *P* = .008; Figure 2F, Table 3). However, progression-free survival did not show any association, with a hazard ratio of 1.37 (95% CI = 0.67 to 2.79; *P* = .37; Supplementary Figure 9B and Supplementary Table 7, available online). In summary, the validation cohort confirmed the prognostic value of CatD established in the ESPAC cohort, highlighting the fact that patients with high CatD expression have an impaired response to gemcitabine treatment.

### Influence of CatD on Gemcitabine Resistance

**Figure 3.**
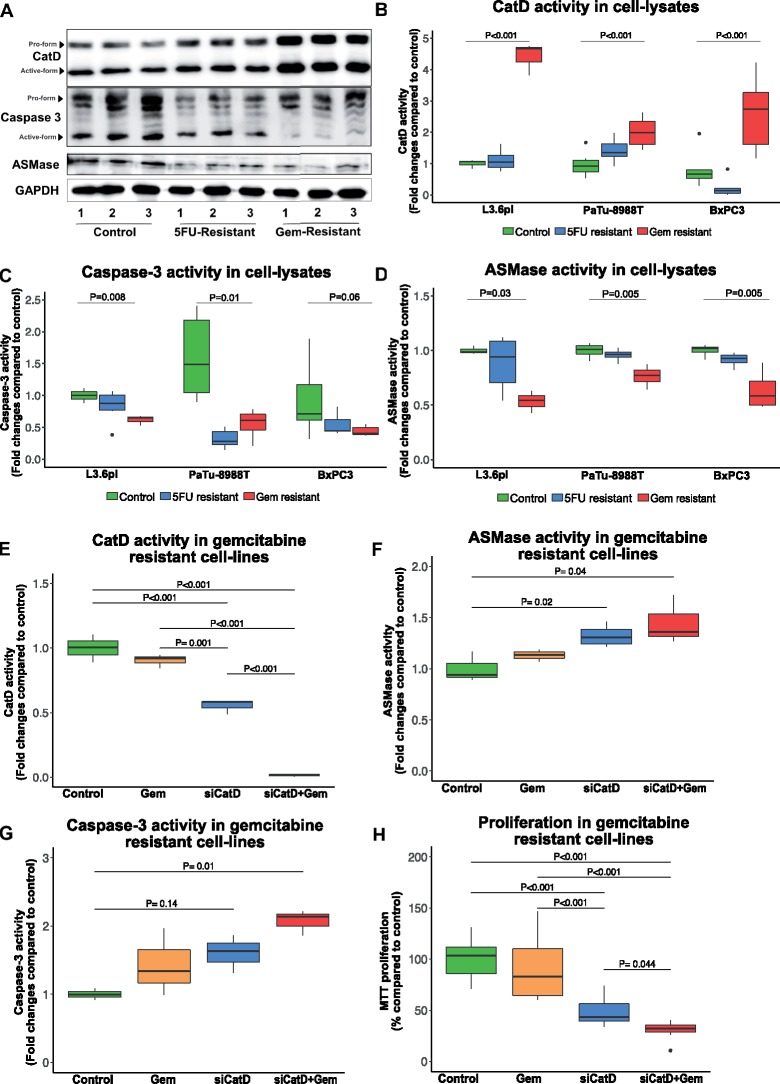
Influence of CatD expression and activity in gemcitabine- and 5FU-resistant cell lines. **A**) Immunoblotting of protein lysates harvested from control, 5FU-resistant and gemcitabine-resistant cell lines for CatD, caspase 3, and ASMase. GAPDH served as loading control. **B–D**) Fluorogenic activity measurement of CatD (**B**), caspase 3 (**C**), and ASMase (**D**) in control, 5FU-resistant, and gemcitabine-resistant cell lines, L3.6pl, PaTu-8988T, and BxPC3. Data represented as fold changes compared to control. **E–G**) Fluorogenic activity measurement of CatD (**E**), caspase 3 (**F**), and ASMase (**G**) on CatD silencing and gemcitabine treatment in gemcitabine-resistant cell line, PaTu-8988T. Data represented as fold changes compared to control. **H**) MTT proliferation assay 48 hours after CatD silencing and gemcitabine treatment in gemcitabine-resistant cell lines. Data represented as percentage change compared to control. All the data are presented as box and whisker plots. *P* < .05 considered statistically significant vs respective controls. 5FU = 5-fluorouracil; ASMase = acid sphingomyelinase; CatD = cathepsin D; GAPDH = gylceraldehyde 3-phosphate dehydrogenase; Gem = gemcitabine; si = small interfering.

The confirmation in the validation cohort showed high CatD expression to be correlated with poor survival in gemcitabine-treated patients. CatD is known to be activated by ceramide, and low levels of ceramide are implicated in gemcitabine resistance (26,27). Acid sphingomyelinase (ASMase) is a rate-limiting enzyme in the conversion of ceramide by breaking down sphingomyelin (28). To study the influence of CatD in driving gemcitabine resistance, we analyzed CatD expression in 5FU- and gemcitabine-resistant cell lines (L3.6pl, BxPC3, and PaTu-8988T). We detected high CatD expression with a concomitant decrease in expression of caspase 3 and ASMase in gemcitabine-resistant cell lines (Figure 3A). 5FU-resistant cell lines did not show a change in CatD expression with respect to control. We confirmed our findings by measuring CatD, caspase 3, caspase 8, caspase 9, and ASMase activity in gemcitabine-resistant and 5FU-resistant cell lines. CatD activity was statistically significantly increased, whereas caspase 3 and ASMase activities were decreased in gemcitabine-resistant cell lines when compared to controls and 5FU-resistant cell lines (Figure 3, B–D; Supplementary Figure 10, available online).

To validate our claims, we used gemcitabine-resistant cell line PaTu-8988T and silenced CatD by small interfering RNA (small interfering CatD). Treatment with gemcitabine (1 µM) in CatD-silenced cells resulted in an increase in ASMase activity. In parallel, we detected an increase in the rate of apoptosis, measured by caspase 3 activity, whereas gemcitabine treatment alone did not result in a statistically significant increase in caspase 3 activity (Figure 3, E–G). Furthermore, silencing of CatD in combination with gemcitabine treatment in gemcitabine-resistant cell lines resulted in a significant decrease in proliferation after 48 hours (Figure 3H). In summary, in a crosstalk between CatD and ASMase, the silencing of CatD in gemcitabine-resistant cell lines renders the cells responsive to gemcitabine treatment.

## Discussion

For this study, we included only patients treated in the ESPAC-1 and ESPAC-3 trials studying the role of adjuvant chemotherapy in pancreatic cancer. With this approach, we aimed for a reduced bias by recruiting a stage-corrected group of patients receiving mono-hemotherapy within a randomized trial to test expression levels of a single biomarker, CatD, in tissues harvested under standardized conditions and externally monitored, quality controlled clinical data. Furthermore, utmost care was taken in generating the tissue microarray to reduce bias of tumor heterogeneity as well as increasing sensitivity and specificity of labeling. The bias of nonrandomized studies precludes separating a predictive therapy–specific effect from a disease prognosis–specific effect. Using patients from retrospective uncontrolled cohorts may have contributed to the variability of previously published studies on the role of CatD (17). Here, we demonstrate the prognostic capacity of CatD expression levels for overall survival in resected PDAC and its potential to predict the efficacy of adjuvant gemcitabine. We could not delineate a correlation between 5FU treatment and CatD expression. However, different prognostic markers (eg, dihydropyridine dehydrogenase) known to be involved could be used in a combinatorial approach to stratify patients (29). These data were confirmed in an independent validation cohort with a prespecified outcome on which sample size calculation was based. Results from both cohorts imply that adjuvant gemcitabine monotherapy might not be considered in patients with high tumor cell CatD expression. Because adjuvant gemcitabine-treated patients with low CatD levels display a survival benefit, low CatD can be an effective predictive marker of efficacy of gemcitabine. Furthermore, in vitro high CatD expression reflected gemcitabine resistance.

Mechanistically, the crosstalk between CatD and ASMase confers resistance to gemcitabine, and if confirmed in a prospective, randomized trial, CatD can be used as either a biomarker in the setting of precision medicine or as a novel drug target for the treatment of pancreatic cancer.

The association of high CatD expression with poor survival is in agreement with previous studies in node-negative breast cancer patients that showed high tumor CatD expression also negatively correlated with survival (12,13,15,30). The one previously published study in which CatD expression in PDAC was explored for possible correlation with metastasis involved 21 patients and showed 81% patients with CatD expression (14). Our study reduces the ambiguity derived from studies that found either prometastatic or antitumorigenic effects associated with CatD expression (9,14,31,32).

In addition to associations with survival outcomes, our study detected an association between CatD expression and gemcitabine responsiveness. One potential explanation regarding how increased CatD expression can influence gemcitabine responsiveness is the crosstalk of CatD with ASMase and ceramide. The balance between bioactive sphingolipids, ceramides, sphingosine, and sphingosine-1-phosphate functions as a biostat with profound effects on cell death, growth, and differentiation (33). It has been shown that ASMase-derived ceramide specifically binds to, and thereby induces, the proteolytic activity of CatD within the endolysosomal compartments (caspase 3 activation; see Figure 3, C and G) (27,34–36). Drug-resistant cells maintain low ceramide levels by increasing sphingomyelin synthesis or by preventing sphingomyelin breakdown (26). Our data suggest that the ceramide–ASMase–CatD axis regulates intracellular ceramide levels by an autocrine loop with reciprocal effects on CatD expression and ASMase activity, and failure of it results in gemcitabine resistance (37).

An obvious drawback of our study is the lack of 5FU-treated patients in our validation cohort. 5FU is not regularly used in clinical care in the adjuvant setting in Germany and not recommended in the German treatment guidelines since the publication of the CONKO-001 study (38) and cross-comparison of efficacy and side effects with the ESPAC-3 data. Currently, FOLFIRINOX (folic acid, 5-fluorouracil, irinotecan, oxaliplatin) would be preferred over a gemcitabine-based regime with respect to the progression-free survival and overall survival data (PRODIGE 24/CCTG PA.6) at the expense of severe adverse effects (39), whereas adjuvant nab-paclitaxel plus gemcitabine did not show improved progression-free survival compared to gemcitabine alone (40). For less-fit patients, gemcitabine plus capecitabine is a treatment of choice (5). Monotherapy with gemcitabine alone is another reasonable option considering its lower toxicity profile, particularly for patients with a borderline performance status or a comorbidity profile that precludes multi-agent therapy (41,42). Furthermore, the COMPASS (43) trial revealed a gemcitabine-based signature to be equally potent in comparison to a platin-based signature, and thus it is still undecided what treatment would be best.

The data presented here comprise the largest and most comprehensive analysis of a prognostic role of CatD on patient survival and support a prognostic as well as predictive role of CatD with regard to therapy response. We believe, subject to prospective validation, in patients with low CatD expression adjuvant treatment with a gemcitabine-backbone therapeutic regimen can be employed, but for patients with high CatD expression, a nongemcitabine regimen such as FOLFORINOX might be of greater benefit. This study could therefore pave the way for introducing CatD expression into the treatment stratification of pancreatic cancer patients provided that randomized, prospective studies can confirm its effectiveness as a biomarker. Furthermore, owing to high expression of CatD in other solid tumors, such as breast cancer and colorectal cancer, CatD can be a predictive marker for therapeutic response in these cancers.

## Funding

This work was supported by the Deutsche Krebshilfe/Dr Mildred-Scheel-Stiftung (109102) and the European Union (EU-FP-7: EPC-TM and EU-FP7-REGPOT-2010–1), PePPP Center of Excellence (MV ESF/14-BM-A55-0045/16; ESF MV V-630-S-150–2012/132/133); and CRU Cancer UK and the Liverpool Cancer Trials Unit Liverpool (DFG SFB-1321, Project A14).

## Notes

Affiliations of authors: Department of Medicine II (UMM, EG, QL, FL, GB, JM), Department of Medicine III (SK, MvBB, VH, SB), Department of Radiation Oncology (CB), University Hospital, LMU-Munich, Munich, Germany; Department of Medicine A, University Medicine Greifswald, Greifswald, Germany (UMM, EL, GB, FUW, MS, MML, JM); National Institute for Health Research Liverpool Pancreas Biomedical Research Centre, University of Liverpool, UK (EC, WG, CH, PG, JPN); Institute of Pathology, Faculty of Medicine, LMU Munich, Munich, Germany (SO, TKi); Leibniz Institute for Prevention Research and Epidemiology – BIPS, Bremen, Germany (MM); Department of General, Visceral, and Tumor Surgery, University Hospital Cologne, Cologne, Germany (YZ, CJB); Department of Community Medicine, University Medicine Greifswald, Greifswald, Germany (TKo); Department of General, Visceral, and Transplant Surgery, Ludwig-Maximilians-University Munich, Munich, Germany (JW, JGD); Department of General, Visceral and Transplantation Surgery, University of Heidelberg, Heidelberg, Germany (JPN, MWB); German Cancer Consortium, German Cancer Research Center, Heidelberg, Germany (TKi, VH, SB).

The authors declare no conflict of interest.

UMM, EL, EG, and JM were involved in the acquisition of the data, analysis, interpretation of the data, and drafting of the manuscript. JM, MML, and UMM were involved in the study design, writing of the manuscript draft, supervision of experimental work, and funding. MM and TKo provided statistical expertise. FL, GB and YZ, CB, and FUW provided experimental expertise. JW, JD, TKo, SB, MvB, CB, and VH provided patients information, tissue, and experimental expertise. WG, EC, JPN, MWB, and CH are principal investigators of ESPAC-3 and ESPAC-TPlus. SO and TKi are expert pathologists.

## Supplementary Material

pkz060_Supplementary_Data
