# Supplementary material for: Cathepsin D Expression and Gemcitabine Resistance in Pancreatic Cancer
Source: JNCI Cancer Spectr. 2019 Aug 16;4(1):pkz060. doi: 10.1093/jncics/pkz060 (PMC7050148; doi:10.1093/jncics/pkz060)
Supplement: pkz060_Supplementary_Data [file pkz060_supplementary_data.pdf]

Cathepsin D expression and gemcitabine resistance in pancreatic cancer

Ujjwal M. Mahajan[1,2], Elisabetta Goni[1], Enno Langhoff[2], Qi Li[1], Eithne Costello[3], William Greenhalf[3], Stephan Kruger[4], Steffen Ormanns[5], Christopher Halloran[3], Paula Ganeh[3], Manuela Marron[6], Felix Lämmerhirt[1], Yue Zhao[7], Georg Beyer[1,2], Frank-Ulrich Weiss[2], Mathias Sendler[2],Christiane J. Bruns[7], Thomas Kohlmann[8], Thomas Kirchner[5,12], Jens Werner[9], Jan G. D'Haese[9], Michael von Bergwelt-Baildon[4], Volker Heinemann[4,12], John P. Neoptolemos[3,10], Markus W. Büchler[10], Claus Belka[11], Stefan Boeck[4,12], Markus M. Lerch[2], Julia Mayerle[1,2#]

[1]*Department of Medicine II, University Hospital, LMU-Munich, Munich, Germany,* [2]*Department of Medicine A, University Medicine Greifswald, Greifswald, Germany*, [3]*NIHR Liverpool Pancreas Biomedical Research Centre, University of Liverpool, UK,* [4]*Department of Medicine III, University Hospital, LMU-Munich,* [5]*Institute of Pathology, Faculty of Medicine, LMU Munich, Munich, Germany,* [6] *Leibniz Institute for Prevention Research and Epidemiology – BIPS, Bremen, Germany,* [7]*Department of General, Visceral and Tumor Surgery, University Hospital Cologne, Cologne, Germany,* [8]*Department of Community Medicine, University Medicine Greifswald, Greifswald, Germany,* [9]Department of General, Visceral, and Transplant Surgery, Ludwig-Maximilians-University Munich, Munich, Germany *,* [10]*Department of General, Visceral and Transplantation Surgery, University of Heidelberg, Heidelberg, Germany,* [11] Department of Radiation Oncology*, University Hospital, LMU-Munich, Munich, Germany,* [12]*DKTK, German Cancer Consortium, German Cancer Research Center (DKFZ), Heidelberg, Germany*

SUPPLEMENTARY METHODS

Study Design

The translational ESPAC-T studies received ethical committee approval for characterization of tumor markers for chemotherapy from the Liverpool (Adult) Research Ethics Committee (07/H1005/87). Use of Good Clinical Practice standard operating procedures[1] ensured a full audit trail and prevented access to outcome data by pathologists and laboratory researchers. After resection for pancreatic ductal adenocarcinoma, patients in the ESPAC-3 study were randomized to receive either 5FU/Folinic acid or gemcitabine. ESPAC-3 was analyzed on an intention to treat basis, but for the ESPAC-T study patients in the treatment arms were selected for inclusion only if treatment was actually received. This study was conducted and reported in accordance with the REMARK criteria[2–4]. A participant variable was regarded as missing if the value of the covariate for the participant is not available and considered as missing completely at random (MCAR) as described previously for complete case analysis[5].

For independent validation, sample size was calculated based on the hazard ratio (HR) in the standard cox PH model of CatD expression calculated from the multivariate analysis in gemcitabine treated arm[6]. The analysis asked for 69 gemcitabine treated patients ensuring a power of 0.8 and $p<0.05$. Considering loss-to follow-up and technical problems in TMA generation, we analysed resected 69 patients with PDAC receiving adjuvant gemcitabine treatment recruited at the University of Munich for validation.

Tissue Microarray Manufacture

The treatment arrays contained cores from patients from ESPAC-3 randomized to either 5FU/Folinic acid or to gemcitabine only. Although fixation protocols for formalin-fixed paraffin embedded tissue blocks varied across centres, Cores were taken from tumor regions identified by an experienced pancreatic pathologist using haematoxylin-eosin stained sections. Tissue microarrays were prepared with two cores from each block, with four to eight cores arrayed for each patient. For all arrays control cores, comprising 3 cores each of colon, kidney, liver, normal pancreas and chronic pancreatitis, were arranged in a fence around the test samples. Each of the treatment was designed as described previously[7]. Sample size for the validation cohort was calculated after completion of analysis of the ESPAC-TPlus cohort.

For the validation cohort, formalin-fixed, paraffin-embedded tumor tissue of **69** randomly selected PDAC cases resected between 2011 and 2017 at the Department of Surgery at the University of Munich was acquired from the archived material at the Department of pathology at LMU. A TMA consisting of three tissue cores of the histologically confirmed tumor tissues, each 1.0 mm in diameter, was constructed using a semiautomatic tissue arrayer (Beecher Instruments, Sun prairie, WI, USA) as described previously[8].

Immunohistochemistry (IHC)

Immunohistochemistry of 2µm sections of the tissue microarrays core for CatD (goat polyclonal CatD, G20, Santacruz, 1:100 diluted in PBS) was performed as described previously[9,10]. Briefly, after deparaffinization and hydration of sections, antigens were retrieved by heat treatment in citrate buffer pH 6.0 (Dako Deutschland GmbH, Hamburg, Germany). Sections were treated with peroxidase block. Slides were incubated with the primary antibody in PBS (as above) at 4^0C overnight. Immunostaining was performed after specific secondary antibodies incubation using DAB complex conjugation techniques (vector Laboratories Ltd., Peterborough, UK). Negative controls were incubated with buffer alone, in place of primary antibody.

Scoring was undertaken according to intensity of cytoplasmic staining in tumor cells ranked from 0 to 3 (0= no staining, 1= low staining, 2= intermediate staining and 3= high staining) and the percentage of tumor cells stained positive was calculated using the algorithm developed for NIH ImageJ software. H-Scores were calculated for each core by multiplying the intensity score by the percentage of tumor cells staining positive and a median H-Score calculated for all cores from each patient. Median H-Score was divided into two cohorts of low (H score ≤ 22.35) and high (H score >22.35) CatD expression.

Cell-lines

The pancreatic adenocarcinoma cell line PaTU-8988T and BxPC3 was obtained by DSMZ, Braunschweig. BxPC3 cells were grown in RPMI-1640 media supplemented with 10% FCS whereas PaTu-8988T cells were grown in DMEM media supplemented with 10% FCS. The gemcitabine sensitive cell lines, BxPC3 and PaTu-8988T has been propagated with increasing drug concentrations of either Gemcitabine or 5FU over an eleven-month period. Gemcitabine was purchased by Lilly, Germany. The initial concentration of both drugs corresponded to a tenth of the IC_{50} value detected prior to starting experiments in the same cell line. After having adapted to the initial drug concentration, the cells were detached by trypsinogen and 0.5 x 10*6 cells were

sub-cultured with the twofold quantity of the previous chemotherapy concentration. In this way, PaTu-8988T cells were adapted up to a concentration of 250nM Gemcitabine or 5FU respectively whereas BxPC3 were adopted to 55nM. All the materials were purchased from Sigma-Aldrich unless otherwise mentioned.

siRNAs

CTSD silencing and validated siRNA (4390824, ID, s137, Lot# ASO0MK0U) targeted against CatD were purchased from Ambion Inc (Thermo Fischer Scientific Brand, Darmstadt, Germany). CatD gene silencing in PaTu-8988T cells were performed by means of electroporation using (Amaxa biosystem) using Amaxa cell line nucleofactor kit V (Lonza, Basel, Switzerland) as per manufacturer's instruction. 10 hours after electroporation, cells were washed with PBS, media was replaced, and cells were treated according to respective experimental conditions.

Western Blot analysis

Plated cancer cell-lines for western blot experiments were homogenised on ice in lysis buffer containing 25 mM HEPES (pH 7.5), 75 mM NaCl, 0.5% Triton X-100, 5% glycerol, 1 mM EDTA in the presence of different protease inhibitors (10 mM NaF, 5 mM Na4P2O7, 1 mM PMFS and 1 mg/ml aprotinin). Protein content was determined by the Bradford assay or BCA kit from Pierce (Rockford, Illinois, USA). In all, 30 µg samples of total protein were loaded on 12.5% polyacrylamide gels and transferred to nitrocellulose membranes for immunoblotting. CatD (Santa Cruz, Heidelburg, Germany, Goat polyclonal CatD, G20 antibody), cleaved caspase-3 (Cell Signalling, Frankfurt am Main, Germany, Rabbit Polyclonal antibody), and ASMase (Abcam, Cambridge, UK, Mouse monoclonal antibody) were used at a dilution of 1:1000, anti-GAPDH (Dunn Labortechnik (Meridian), Asbach, Germany, Mouse monoclonal, clone 6C5) was used as loading control. Densitometry for obtained was performed using Image J software and dividing optical density (OD) of bands by OD of GAPDH band after background subtraction.

Proliferation assay

To assess the influence on proliferation in gemcitabine resistant cells, Gemcitabine resistant cells were PaTu-8988T cells were treated with either of gemcitabine, silencing by siRNA targeted against CatD and silencing by siRNA targeted against CatD followed by gemcitabine treatment after 6 hours. Untreated cells served as a control. Cells proliferation were monitored using MTT proliferation assay at 48 hours. Briefly, after completion of treatment, cells were treated with 20µl of 2.5mg/ml of MTT in PBS for 2 hours and cells were lysed afterward using 150µl of DMSO and plates were measured by photometric assay at room temperature with a Spectramax spectrophotometer (BD biosciences, Heidelberg, Germany) at 595 nm. Obtained results were normalized for maximum proliferation for negative control at 48 hours.

Enzymatic activity estimation

Purified Cathepsin D (Cathepsin D) enzyme (Calbiochem, EMD Biosciences, Inc. CA, USA) was used as standard. CatD activity in cell lysates and media supernatant were determined by fluorometric enzyme kinetic over 30 min at 37°C using Cathepsin D substrate (Enzo Lifesciences GmbH, Loerrach, Germany) measured at ex=340nm and em=420nm using FluorStar Optima Flourometer (BMG LabTech, Ortenberg, Germany). Cell lysates were resuspended in 100mM Sodium acetate containing 5mM $CaCl_2$ at pH 4.0 sonicated and supernatants were used for enzymatic activity detection. Caspases activities were measured using the fluorogenic substrate Ac-DEVD-AMC (BD Biosciences, Sane Jose, USA, substrate for caspase 3), fluorogenic substrate Ac-LEHD-AMC (BD Biosciences, Sane Jose, USA, substrate for caspase 8) and fluorogenic substrate Ac-IETD-AMC (BD Biosciences, Sane Jose, USA, substrate for caspase 9) as per manufacturers' instruction. ASMase activity was measured using the HMU-PC substrate (6, hexadecanoylamino-4-methylumbelliferyl-phpsphorylcholine, Moscardam substrates (Carbosynth), Berkshire, UK) as per manufacturers' instruction. Arbitrary fluorescent units were normalized by protein content of respective samples.

Statistical Analysis

The first null hypothesis (H_0) was that CatD levels do not predict survival in patients receiving adjuvant chemotherapy. All analyses were carried out using using R 3.4.4 GUI 1.70 El Capitan build (7507) and R-studio 1.1.442 on an intention to treat basis, retaining patients in their randomized treatment groups and including non-eligible patients. For adjuvant chemotherapy, Patients had to be fully recovered from the operation, with a WHO performance score of 2 or lower and a life expectancy of more than 3 months. Overall survival was measured from the date of resection to date of death from any cause. Patients remaining alive were censored at the date last seen alive. Progression-free survival was measured from date of resection to date of death from any cause or date of local tumor recurrence or metastases. Patients remaining alive and progression-free were censored at the date last seen alive as per ESPAC-3 trial[11].Survival estimates were analyzed by the method of Kaplan-Meier; differences between groups were assessed using the Mantel-Cox log rank test. Multivariate Cox regression analyses were used to adjust the overall treatment effect by all important prognostic variables on a complete case basis. Covariates were included in the multivariable model using a forward stepwise regression approach based on the Akaike Information Criterion if they had an unadjusted log-rank significance of $P<0.25$. The Cox proportional hazards assumption was checked using scaled Schoenfeld residuals. Initial exploratory analyses showed that the time to the start of treatment had a different effect depending on whether or not a patient completed therapy, which was therefore included as a nested effect. The r packages used for the analysis were: survival, survminer (for survival analysis and Cox proportional hazard model), stepp (for sub-population treatment effect pattern plot), bhm (for predictive and prognostic biomarker treatment effect using biomarkers threshold), Hmisc (for multiple imputation for the missing values), NPHMC (for sample size calculation using hazard ratio) and ggplot2, ggpubr (for plotting). A two-sided significance of $P<0.05$ was used throughout.

All data from cell-culture experiments are expressed as Box and whiskers plot (with 25% quartile, median and 75% quartile) from at least four individual experiments in each group. One-way ANOVA followed by Tukey's post-hoc analysis was used for statistical evaluation of all the *in-vitro* data.

REFERENCES FOR SUPPLEMENATARY METHODS

1. Sarzotti-Kelsoe M, Cox J, Cleland N, et al. Evaluation and Recommendations on Good Clinical Laboratory Practice Guidelines for Phase I–III Clinical Trials. *PLoS Med*. 2009;6(5):e1000067. doi:10.1371/journal.pmed.1000067

2. Greenhalf W, Ghaneh P, Neoptolemos JP, et al. Pancreatic Cancer hENT1 Expression and Survival From Gemcitabine in Patients From the ESPAC-3 Trial. *JNCI J Natl Cancer Inst*. 2014;106(1):djt347-djt347. doi:10.1093/jnci/djt347

3. Altman DG, McShane LM, Sauerbrei W, Taube SE. Reporting Recommendations for Tumor Marker Prognostic Studies (REMARK): explanation and elaboration. *PLoS Med*. 2012;9(5):e1001216. doi:10.1371/journal.pmed.1001216

4. McShane LM, Altman DG, Sauerbrei W, Taube SE, Gion M, Clark GM. REporting recommendations for tumour MARKer prognostic studies (REMARK). *Br J Cancer*. 2005;93(4):387-391. doi:10.1038/sj.bjc.6602678

5. Ibrahim JG, Chu H, Chen M-H. Missing Data in Clinical Studies: Issues and Methods. *J Clin Oncol*. 2012;30(26):3297-3303. doi:10.1200/JCO.2011.38.7589

6. Cai C, Wang S, Lu W, Zhang J. NPHMC: An R-package for estimating sample size of proportional hazards mixture cure model. *Comput Methods Programs Biomed*. 2014;113(1):290-300. doi:10.1016/j.cmpb.2013.10.001

7. Greenhalf W, Ghaneh P, Neoptolemos JP, et al. Pancreatic Cancer hENT1 Expression and Survival From Gemcitabine in Patients From the ESPAC-3 Trial. *JNCI J Natl Cancer Inst*. 2014;106(1):djt347-djt347. doi:10.1093/jnci/djt347

8. Ormanns S, Assmann G, Reu S, et al. ALK expression is absent in pancreatic ductal adenocarcinoma. *J Cancer Res Clin Oncol*. 2014;140(9):1625-1628. doi:10.1007/s00432-014-1774-4

9. Mahajan UM, Teller S, Sendler M, et al. Tumour-specific delivery of siRNA-coupled superparamagnetic iron oxide nanoparticles, targeted against PLK1, stops progression of pancreatic cancer. *Gut*. May 2016. doi:10.1136/gutjnl-2016-311393

10. Mahajan UM, Langhoff E, Goni E, et al. Immune Cell and Stromal Signature Associated with Progression-free Survival of Patients with Resected Pancreatic Ductal Adenocarcinoma. *Gastroenterology*. August 2018. doi:10.1053/j.gastro.2018.08.009

11. Neoptolemos JP, Stocken DD, Bassi C, et al. Adjuvant chemotherapy with fluorouracil plus folinic acid vs gemcitabine following pancreatic cancer resection: a randomized controlled trial. *JAMA*. 2010;304(10):1073-1081. doi:10.1001/jama.2010.1275

Supplementary Table 1: Relationship between Tumor cells CatD levels and patients or Tumor characteristics.

Characteristics		CatD				CatD-5FU				CatD-GEM			
		Low CatD	*High CatD*	*Total*	*P*	*Low CatD*	*High CatD*	*Total*	*P*	*Low CatD*	*High CatD*	*Total*	*P*
**Baseline Performance Scale**	**0**	26	95	121 (33.4%)	*0.85*	19	42	61 (35.1%)	*0.48*	7	53	60 (31.9%)	*0.92*
	**1**	45	150	195 (53.9%)		34	56	90 (51.7%)		11	94	105 (55.9%)	
	**2**	9	37	46 (12.7%)		6	17	23 (13.2%)		3	20	23 (12.2%)	
**Maximum Tumor Diameter**	**<30mm**	49	160	209 (61.1%)	*0.29*	38	72	110 (66.7%)	*0.77*	11	88	99 (55.9%)	*0.89*
	**≥30mm**	24	109	*133 (38.9%)*		17	38	*55 (33.3%)*		7	71	*78 (44.1%)*	
**Gender**	**Female**	36	117	153 (42.3%)	*0.66*	26	49	75 (43.1%)	*0.98*	10	68	78 (41.5%)	*0.71*
	**Male**	44	165	209 (57.7%)		33	66	99 (56.9%)		11	99	110 (58.5%)	
**Tumor Grade**	**Well**	5	22	27 (7.6%)	*0.79*	4	8	12 (7.0%)	*0.69*	1	14	15 (8.2%)	*0.64*
	**Moderate**	50	179	229 (64.5%)		35	73	108 (63.2%)		15	106	121 (65.8%)	
	**Poor**	24	75	99 (27.9%)		20	31	51 (29.8%)		4	44	48 (26.1%)	
**Tumor Stage**	**1**	4	19	23 (6.4%)	*0.47*	3	9	12 (6.9%)	*0.23*	1	10	11 (5.9%)	*0.67*
	**2**	27	71	98 (27.4%)		21	26	47 (27.2%)		6	45	51 (27.6%)	
	**3**	46	182	228 (63.7%)		33	75	108 (62.4%)		13	107	120 (64.9%)	
	**4**	2	7	9 (2.5%)		1	5	6 (3.5%)		1	2	3 (1.6%)	
**Lymph Node Status**	**Negative**	23	56	79 (21.8%)	*0.12*	19	19	38 (21.8%)	*0.02*	4	37	41 (21.8%)	*0.92*
	**Positive**	57	226	283 (78.2%)		40	96	136 (78.2%)		17	130	147 (78.2%)	
**Resection Margin**	**Negative**	45	157	202 (55.8%)	*0.97*	31	59	90 (51.7%)	*0.99*	14	98	112 (59.6%)	*0.64*
	**Positive**	35	125	160 (44.2%)		28	56	84 (48.3%)		7	69	76 (40.4%)	
**Diabetes**	**No**	62	212	274 (78.7%)	*0.37*	44	87	131 (78.9%)	*0.40*	18	125	143 (78.6%)	*0.41*
	**IDDM**	6	37	43 (12.4%)		5	15	20 (12.0%)		1	22	23 (12.6%)	
	**NIDDM**	8	23	31 (8.9%)		7	8	15 (9.0%)		1	15	16 (8.8%)	
**Smoke**	**Never**	38	105	143 (43.9%)	*0.10*	29	43	72 (45.6%)	*0.21*	9	62	71 (42.3%)	*0.60*
	**Past**	24	103	127 (39.0%)		18	42	60 (38.0%)		6	61	67 (39.9%)	
	**Present**	8	48	56 (17.2%)		6	20	26 (16.5%)		2	28	30 (17.9%)	
**Local Invasion**	**No**	35	154	189 (53.7%)	*0.21*	31	61	92 (54.1%)	*0.94*	4	93	97 (53.3%)	*<0.01*
	**Yes**	40	123	163 (46.3%)		25	53	78 (45.9%)		15	70	85 (46.7%)	
**Age (Years)**	**<64**	35	153	188 (51.9%)	*0.12*	28	64	92 (52.9%)	*0.38*	7	89	96 (51.1%)	*0.13*
	**≥64**	45	129	174 (48.1%)		31	51	82 (47.1%)		14	78	92 (48.9%)	
**Post Op CA19-9 (Units)**	**<27**	34	101	135 (50.9%)	*0.38*	26	34	60 (45.8%)	*0.20*	8	67	75 (56.0%)	*0.63*
	**≥27**	26	104	130 (49.1%)		22	49	71 (54.2%)		4	55	59 (44.0%)	

Supplementary Table 2: LIFETEST procedures of the overall and progression free survival factors

CatD High/Low Expression Tumor cells							
		Overall Survival			Progression free survival		
**Strata Comparison**		**m(st) (95% CI)**	χ^2	***p***	**m(pfs) (95% CI)**	χ^2	***p***
5FU-High Vs 5FU-Low	5FU-Low<br>5FU-High	25.25 (17.44-30.39)<br>21.45 (15.90-27.49)	1.30	0.26	14.55 (9.95-20.10)<br>12.81 (9.88-15.34)	0.20	0.62
5FU-High Vs GEM-High	5FU-High<br>GEM-High	21.45 (15.90-27.49)<br>20.97 (16.68-25.13)	<0.01	0.97	12.81 (9.88-15.34)<br>12.00 (10.51-14.19)	0.23	0.62
5FU-High Vs GEM-Low	5FU-High<br>GEM-Low	21.45 (15.90-27.49)<br>31.24 (24.11-51.31)	3.11	0.07	12.81 (9.88-15.34)<br>19.25 (12.74-26.67)	1.69	0.20
5FU-Low Vs GEM-High	5FU-Low<br>GEM-High	25.25 (17.44-30.39)<br>20.97 (16.68-25.13)	1.53	0.21	14.55 (9.95-20.10)<br>12.00 (10.51-14.19)	0.73	0.39
5FU-Low Vs GEM-Low	5FU-Low<br>GEM-Low	25.25 (17.44-30.39)<br>31.24 (24.11-51.31)	1.26	0.26	14.55 (9.95-20.10)<br>19.25 (12.74-26.67)	0.94	0.33
GEM-High Vs GEM-Low	GEM-Low<br>GEM-High	31.24 (24.11-51.31)<br>20.97 (16.68-25.13)	3.58	0.05	19.25 (12.74-26.67)<br>12.00 (10.51-14.19)	2.88	0.08

Supplementary Table 3: Univariate analysis of progression free survival factors.

	Hazard Ratio (95% CI)		
**Characteristics**		**5-Fluorouracil/Folinic acid**	**Gemcitabine**
**Age**	**N=362**	**N=174**	**N=188**
	0.99 (0.97 to 1.00)	0.98 (0.97 to 1.00)	0.99 (0.97 to 1.01)
	χ^2=2.28 (P=0.13)	χ^2=1.69 (P=0.19)	χ^2=0.66 (P=0.41)
**Sex**	**N=362**	**N=174**	**N=188**
Female	1(Referent)	1(Referent)	1(Referent)
Male	0.86 (0.68 to 1.08)	0.86 (0.62 to 1.19)	0.86 (0.63 to 1.18)
	χ^2=1.61 (P=0.20)	χ^2=0.77 (P=0.38)	χ^2=0.85 (P=0.35)
**Smoking**	**N=326**	**N=158**	**N=168**
Never	1(Referent)	1(Referent)	1(Referent)
Past	1.06 (0.81 to 1.37)	1.06 (0.73 to 1.55)	1.07 (0.74 to 1.54)
	χ^2=2.79 (P=0.67)	χ^2=1.06 (P=0.73)	χ^2=2.02 (P=0.70)
Present	1.32 (0.95 to 1.82)	1.28 (0.80 to 2.04)	1.38 (0.87 to 2.18)
	χ^2=2.00 (P=0.09)	χ^2=2.00 (P=0.30)	χ^2=1.97 (P=0.15)
**Lymph node invasion**	**N=362**	**N=174**	**N=188**
Negative	1(Referent)	1(Referent)	1(Referent)
Positive	1.94 (1.44 to 2.59)	2.28 (1.48 to 3.50)	1.67 (1.12 to 2.48)
	χ^2=19.88 (P<0.001)	χ^2=14.14 (P<0.001)	χ^2=6.41 (P=0.01)
**Resection margin**	**N=362**	**N=174**	**N=188**
Negative	1(Referent)	1(Referent)	1(Referent)
Positive	1.59 (1.26 to 1.99)	1.65 (1.18 to 2.30)	1.53 (1.12 to 2.10)
	χ^2=15.97 (P<0.001)	χ^2=8.818 (P=0.01)	χ^2=7.17 (P=0.01)
**Local invasion**	**N=352**	**N=170**	**N=182**
No	1(Referent)	1(Referent)	1(Referent)
Yes	1.35 (1.07 to 1.69)	1.36 (0.98 to 1.89)	1.31 (0.96 to 1.80)
	χ^2=6.80 (P=0.01)	χ^2=3.41 (P=0.06)	χ^2=2.98 (P=0.08)
**Tumor stage**	**N=358**	**N=173**	**N=185**
I	1(Referent)	1(Referent)	1(Referent)
II	1.51 (0.87 to 2.62)	1.46 (0.68 to 3.15)	1.51 (0.67 to 3.35)
	χ^2=10.79 (P=0.14)	χ^2=7.46 (P=0.32)	χ^2=3.01 (P=0.31)
III	1.98 (1.17 to 3.35)	2.14 (1.03 to 4.44)	1.81 (0.84 to 3.90)
	χ^2=3.00 (P=0.01)	χ^2=3.00 (P=0.04)	χ^2=3.00 (P=0.12)
IV	1.13 (0.45 to 2.76)	1.33 (0.43 to 4.07)	0.84 (0.17 to 4.09)
	χ^2=0.01 (P=0.79)	χ^2=0.05 (P=0.62)	χ^2=0.26 (P=0.83)
**Post Operative CA19-9**	**N=265**	**N=131**	**N=134**
	1.00 (1.00 to 1.00)	1 (1.00 to 1.00)	1 (1.00 to 1.00)
	χ^2=13.61 (P<0.001)	χ^2=6.6 (P=0.01)	χ^2=8.8 (P<0.001)
**Maximum Tumor size**	**N=342**	**N=165**	**N=177**
	1.00 (0.99 to 1.00)	1 (0.99 to 1.00)	1 (0.99 to 1.01)
	χ^2=2.13 (P=0.14)	χ^2=0.97 (P=0.32)	χ^2=1.91 (P=0.17)
**Differentiation status**	**N=355**	**N=171**	**N=184**
Well	1(Referent)	1(Referent)	1(Referent)
Moderate	0.90 (0.58 to 1.37)	0.86 (0.44 to 1.65)	0.9 (0.51 to 1.58)
	χ^2=1.94 (P=0.61)	χ^2=0.22 (P=0.65)	χ^2=4.20 (P=0.72)
Poor	1.07 (0.68 to 1.68)	0.84 (0.42 to 1.69)	1.31 (0.72 to 2.41)
	χ^2=2.00 (P=0.77)	χ^2=0.21 (P=0.64)	χ^2=2.00 (P=0.37)
**CatD expression**	**N=362**	**N=174**	**N=188**
Low	1(Referent)	1(Referent)	1(Referent)
High	1.22 (0.92 to 1.59)	1.08 (0.77 to 1.53)	1.55 (0.93 to 2.61)
	χ^2=2.00 (P=0.16)	χ^2=0.23 (P=0.63)	χ^2=2.84 (P=0.09)

Supplementary Table 4: Multivariate analysis of Overall survival factors excluding CatD

	ESPAC-Tplus cohort								Validation cohort			
Covariates	**5FU/FA (n=158)**				**Gemcitabine (n=167)**				**Gemcitabine (n=63)**			
	HR	***(95% CI)***	***z-stat***	***P***	***HR***	***(95% CI)***	***z-stat***	***P***	***HR***	***(95% CI)***	***z-stat***	***P***
**Resection Margin**												
Negative		1.00 (Referent)				1.00 (Referent)				1.00 (Referent)		
Positive	1.63	1.14 to 2.34	2.67	**0.007**	1.29	0.91 to 1.83	1.48	0.13	1.08	0.44 to 2.64	0.16	0.86
**Smoking status**												
Never		1.00 (Referent)				1.00 (Referent)				--		
Past	0.87	0.58 to 1.29	-0.67	0.50	1.29	0.88 to 1.88	1.14	0.18				
Present	0.96	0.59 to 1.60	-0.10	0.91	1.67	1.04 to 2.69	2.11	**0.03**				
**Lymph node status**												
Negative		1.00 (Referent)				1.00 (Referent)				1.00 (Referent)		
Positive	2.22	1.34 to 3.67	3.12	**0.001**	1.62	1.03 to 2.54	2.10	**0.03**	0.94	0.43 to 2.03	-0.15	0.89
**Local invasion**												
Negative		1.00 (Referent)				1.00 (Referent)				1.00 (Referent)		
Positive	1.41	0.98 to 2.02	1.89	0.05	1.31	0.92 to 1.86	1.55	0.12	1.37	0.67 to 2.81	0.88	0.37
**C-statistic**		**0.62 ± 0.02**				**0.59 ± 0.02**				**0.59 ± 0.05**		
**AIC**		**1088.01**				**1202.22**				**248.55**		
**Likelihood ratio test**		**25.74 (P<0.001)**				**15.24 (P=0.009)**				**0.85 (P=0.80)**		

Supplementary Table 5: Multivariate analysis of CatD with progression free survival factors

	ESPAC-Tplus cohort								Validation cohort			
Covariates	**5FU/FA (n=158)**				**Gemcitabine (n=167)**				**Gemcitabine (n=61)**			
	HR	***(95% CI)***	***z-stat***	***P***	***HR***	***(95% CI)***	***z-stat***	***P***	***HR***	***(95% CI)***	***z-stat***	***P***
**Resection Margin**												
Negative		1.00 (Referent)				1.00 (Referent)				1.00 (Referent)		
Positive	1.52	1.07 to 2.16	2.35	**0.01**	1.35	0.96 to 1.89	1.77	0.07	0.81	0.35 to 1.84	-0.49	0.62
**Smoking status**												
Never		1.00 (Referent)				1.00 (Referent)				--		
Past	0.84	0.57 to 1.24	-0.84	0.39	1.06	0.73 to 1.53	0.33	0.74				
Present	1.14	0.70 to 1.84	0.50	0.58	1.47	0.92 to 2.36	1.62	0.10				
**Lymph node status**												
Negative		1.00 (Referent)				1.00 (Referent)				1.00 (Referent)		
Positive	2.26	1.40 to 3.65	3.37	**<0.001**	1.54	1.00 to 2.35	1.99	**0.04**	1.87	0.87 to 3.98	1.62	0.10
**Local invasion**												
Negative		1.00 (Referent)				1.00 (Referent)				1.00 (Referent)		
Positive	1.37	0.97 to 1.94	1.80	0.07	1.55	1.10 to 2.19	2.50	**0.01**	1.16	0.60 to 2.23	0.45	0.64
**CatD expression**												
Low		1.00 (Referent)				1.00 (Referent)				1.00 (Referent)		
High	0.86	0.59 to 1.26	-0.74	0.45	1.94	1.08 to 3.49	2.21	**0.02**	1.37	0.67 to 2.79	0.88	0.37
**C-statistic**	**0.61 ± 0.02**				**0.60 ± 0.02**				**0.57 ± 0.04**			
**AIC**	**1152.07**				**1260.37**				**306.53**			
**Likelihood ratio test**	**26.39 (P<0.001)**				**20.64 (P=0.002)**				**4.08 (P=0.40)**			
Comparison with model Leaving the CatD Expression	**χ^2= 0.55, P=0.45**				**χ^2= 5.74, P=0.01**				**χ^2= 0.81, P=0.36**			

Supplementary Table 6: Demographics, surgery and pathology features of the complete cases in ESPAC cohort

Demographics		Total	Chemotherapy		Overlap of Total (Percent of original data)
Characteristic			5FU/FA	Gemcitabine	
		N=287	N=139	N=148	
Age Median (IQR) years		63 (32-83)	59 (38-83)	64 (32-82)	79.28%
Sex	Female	125 (43.55%)	70(42.44%)	66 (44.59%)	81.70%
	Male	162 (56.44%)	99 (57.55%)	82 (55.40%)	77.51%
Baseline Performance Score	0	97 (33.79%)	48 (34.53%)	49 (33.10%)	80.17%
	1	154 (53.65%)	72 (51.79%)	82 (55.40%)	78.97%
	2	36 (12.54%)	19 (13.66%)	17 (11.48%)	78.26%
Diabetic	No	219 (76.30%)	106 (76.25%)	113 (76.35%)	79.93%
	NIDDM	29 (10.10%)	14 (10.07%)	15 (10.13%)	93.55%
	IDDM	39 (13.55%)	19 (13.66%)	20 (13.51%)	90.70%
Smoking	Never	125 (43.55%)	63 (45.32%)	62 (41.89%)	87.41%
	Past	116 (40.41%)	55 (39.56%)	61 (41.21%)	91.34%
	Present	46 (16.02%)	21 (15.10%)	25 (16.89%)	82.14%
Surgery to Randomisation Median (IQR) days		49 (4-92)	49 (4-88)	49 (7 -92)	79.28%
Surgery	Whipples resection	147 (51.21%)	76 (54.67%)	71 (47.97%)	79.46%
	Pylorus preserving	114 (39.72%)	50 (35.97%)	64 (43.24%)	82.01%
	Distal pancratectomy	17 (5.92%)	7 (5.03%)	10 (6.75%)	89.47%
	Total pancreatectomy	9 (3.13%)	6 (4.31%)	3 (2.02%)	81.82%
Extent of resection	Standard	216 (75.26%)	110 (79.13%)	106 (71.62%)	82.44%
	Radical	40 (13.93%)	18 (12.94%)	22 (14.86%)	85.11%
	Extended Radical	31 (10.80%)	11 (7.91%)	20 (13.51%)	83.78%
Tumor Grade	Well	22 (7.66%)	9 (6.47%)	13 (8.78%)	81.48%
	Moderate	184 (64.11%)	88 (63.30%)	96 (64.86%)	80.35%
	Poor	81 (28.22%)	42 (30.21%)	39 (26.35%)	81.82%
Lymph Node Invasion	Negative	64 (22.29%)	31 (22.30%)	33 (22.29%)	81.01%
	Positive	223 (77.70%)	108 (77.69%)	115 (77.70%)	78.80%
Resection Margin	Negative	162 (56.44%)	72 (51.79%)	90 (60.81%)	80.20%
	Positive	125 (43.55%)	67 (48.20%)	58 (39.19%)	78.13%
Local Invasion	No	155 (54.00%)	75 (53.95%)	80 (54.05%)	82.01%
	Yes	132 (46.00%)	64 (46.04%)	68 (49.94%)	80.98%
Tumor stage	I	16 (5.57%)	8 (5.75%)	8 (5.40%)	69.57%
	II	83 (28.91%)	40 (28.77 %)	43 (29.05%)	84.69%
	III	181 (63.06%)	86 (61.87%)	95 (64.18%)	79.39%
	IV	7 (2.43%)	5 (3.59%)	2 (1.35%)	77.78%
CatD expression	Low	61 (21.25%)	46 (33.09%)	15 (10.13%)	76.25%
	High	226 (78.75%)	93 (66.90%)	133 (89.86%)	80.14%

Supplementary Table 7: Multivariate analysis of complete cases in ESPAC-Tplus cohort for CatD with overall survival factors

	ESPAC-Tplus cohort							
Covariates	5FU/FA (n=139)				Gemcitabine (n=148)			
	HR	*(95% CI)*	*z-stat*	*P*	*HR*	*(95% CI)*	*z-stat*	*P*
**Resection Margin**								
Negative		1.00 (Referent)				1.00 (Referent)		
Positive	1.63	1.10 to 2.41	2.47	**0.01**	1.25	0.86 to 1.82	1.18	0.23
**Smoking status**								
Never		1.00 (Referent)				1.00 (Referent)		
Past	0.94	0.61 to 1.44	-0.26	0.79	1.11	0.74 to 1.68	0.52	0.60
Present	1.06	0.60 to 1.85	0.20	0.83	1.59	0.95 to 2.66	1.79	0.07
**Lymph node status**								
Negative		1.00 (Referent)				1.00 (Referent)		
Positive	2.35	1.38 to 4.02	3.14	**0.001**	1.48	0.91 to 2.40	1.61	0.10
**Local invasion**								
Negative		1.00 (Referent)				1.00 (Referent)		
Positive	1.40	0.94 to 2.08	1.69	0.08	1.65	1.12 to 2.43	2.55	**0.01**
**CatD expression**								
Low		1.00 (Referent)				1.00 (Referent)		
High	0.92	0.61 to 1.40	-0.34	0.72	2.24	1.14 to 4.40	2.35	**0.01**
**C-statistic**		**0.64 ± 0.03**				**0.63 ± 0.02**		
**AIC**		**914.31**				**1016.83**		
**Likelihood ratio test**		**25.49 (P<0.001)**				**19.59 (P=0.003)**		
Comparison with model Leaving the CatD Expression		**χ^2= 0.12, P=0.72**				**χ^2= 6.63, P=0.01**		

Supplementary Table 8: Demographics, surgery and pathology features of the patients scored for CatD in validation cohort.

Demographics<br>Characteristic		Total
		**N=69**
**Age Median (IQR) years**		67(40-83)
**Sex**	**Female**	30 (43.4%)
	**Male**	39 (56.5%)
**ECOG**		**N=65**
	**0**	26 (55.3%)
	**1**	18 (38.2%)
	**2**	3 (6.3%)
**Diabetic status**		**N=69**
	**No**	51 (78.4%)
	**IDDM (Type 1)**	5 (7.6%)
	**NIDDM (Type 2)**	8 (12.3%)
	**Type 3 DM**	1 (1.5%)
**Post-Op. CA 19-9 Median (IQR) KU/l**		**N=59**<br>96 (0-7950)
**Tumor grade**		**N=66**
	**Well**	1 (1.5%)
	**Moderate**	20 (30.3%)
	**Poor**	45 (68.1%)
**Lymph Node invasion**		**N=66**
	**Negative**	20 (30.3%)
	**Positive**	46 (69.6%)
**Resection margin**	**Negative**	56 (81.1%)
	**Positive**	13 (18.8%)
**Local invasion**		**N=66**
	**No**	37 (56.0%)
	**Yes**	29 (43.9%)
**Tumor stage**		**N=66**
	**I**	2 (3.0%)
	**II**	5 (7.5%)
	**III**	57 (86.3%)
	**IV**	2 (3.7%)
**Perineural invasion**		**N=66**
	**No**	18 (27.3%)
	**Yes**	48 (72.7%)
**CatD expression**	**Low**	19 (27.5%)
	**High**	50 (72.5%)

Supplementary Table 9: Relationship between Tumor cells CatD levels and patients or Tumor characteristics in validation cohort.

		CatD- GEM validation cohort			
**Characteristics**		***Low CatD***	***High CatD***	***Total***	***P***
**ECOG**	**0**	8	18	26	*0.98*
	**1**	6	12	18	
	**2**	1	2	3	
**Gender**	**Female**	11	19	30	*0.22*
	**Male**	8	31	39	
**Tumor grade**	**Well**	1	0	1	*0.09*
	**Moderate**	8	12	20	
	**Poor**	10	35	45	
**Tumor stage**	**I**	0	2	2	*0.66*
	**II**	2	3	5	
	**III**	16	41	57	
	**IV**	1	1	2	
**Lymph node status**	**Negative**	3	17	20	*0.18*
	**Positive**	16	30	46	
**Resection margin**	**Negative**	17	39	56	*0.45*
	**Positive**	2	11	13	
**Diabetes**	**No**	16	35	51	*0.61*
	**IDDM**	1	4	5	
	**NIDDM**	1	7	8	
	**Type 3**	0	1	2	
**Local Invasion**	**No**	10	27	37	*0.93*
	**Yes**	9	20	29	
**Age (Years)**	**<67**	13	33	36	*0.99*
	**≥67**	6	17	23	
**Post Op CA19-9 (Units)**	**<109**	10	15	25	*0.03*
	**≥109**	4	29	33	
**Perineural invasion**	**No**	7	11	18	*0.42*
	**Yes**	12	36	48	

Supplementary Table 10: Univariate analysis of overall and progression free survival factors in validation cohort.

Gem-Validation cohort: Univariate analysis			
**Characteristics**		**OS**	**PFS**
**Sex**		**N=69**	**N=67**
	Female	1(Referent)	1(Referent)
	Male	1.13 (0.59 to 2.17)<br>χ^2=0.15 (P=0.69)	1.25 (0.69 to 2.25)<br>χ^2=0.55 (P=0.45)
**Lymph node invasion**		**N=66**	**N=64**
	Negative	1(Referent)	1(Referent)
	Positive	0.99 (0.49 to 1.99)<br>χ^2=0.01 (P=0.99)	1.73 (0.89 to 3.36)<br>χ^2=2.68 (P=0.10)
**Resection margin**		**N=69**	**N=67**
	Negative	1(Referent)	1(Referent)
	Positive	1.11 (0.49 to 2.55)<br>χ^2=0.07 (P=0.78)	0.98 (0.47 to 2.05)<br>χ^2=0.01 (P=0.96)
**Tumor grade**		**N=66**	**N=64**
	Poor	1(Referent)	1(Referent)
	Moderate	0.04 (0.19 to 0.89)<br>χ^2=2.00 **(P=0.02)**	0.05 (0.26 to 0.99)<br>χ^2=2.00 **(P=0.04)**
	Well	<0.001 (0 to Inf)<br>χ^2=5.11 (P=0.99)	<0.001 (0 to Inf)<br>χ^2=3.84 (P=0.99)
**Local invasion**		**N=66**	**N=64**
	No	1(Referent)	1(Referent)
	Yes	1.29 (0.66 to 2.50)<br>χ^2=0.58 (P=0.44)	1.24 (0.68 to 2.23)<br>χ^2=0.52 (P=0.47)
**Tumor stage**		**N=66**	**N=64**
	I	1(Referent)	1(Referent)
	II	0.09 (0.01 to 0.75)<br>χ^2=13.21 **(P=0.02)**	0.02 (0.00 to 0.40)<br>χ^2=8.99 **(P=0.01)**
	III	0.04 (0.00 to 0.26)<br>χ^2=3.00 **(P<0.01)**	0.01 (0.00 to 0.25)<br>χ^2=3.00 **(P<0.01)**
	IV	0.02 (0.00 to 0.59)<br>χ^2=0.01 **(P<0.01)**	0.00 (0.00 to 0.29)<br>χ^2=0.02 **(P<0.01)**
**Perineural invasion**		**N=66**	**N=64**
	Negative	1 (Referent)	1 (Referent)
	Positive	2.16 (0.94 to 4.98)<br>χ^2=3.30 (P=0.06)	2.24 (1.03 to 4.87)<br>χ^2=4.87 **(P=0.02)**
**Post-operative CA19.9**		**N=59**<br>1.00 (1.00 to 1.00)<br>χ^2=1.74 (P=0.18)	**N=57**<br>1.00 (1.00 to 1.00)<br>χ^2= 10.67 **(P<0.01)**
**CatD expression**		**N=69**	**N=67**
	Low	1(Referent)	1(Referent)
	High	3.04 (1.28 to 7.24)<br>χ^2=6.35 **(P=0.01)**	1.17 (0.60 to 2.30)<br>χ^2=0.22 **(P=0.63)**

SUPPLEMENTARY FIGURES

Supplementary Figure 1

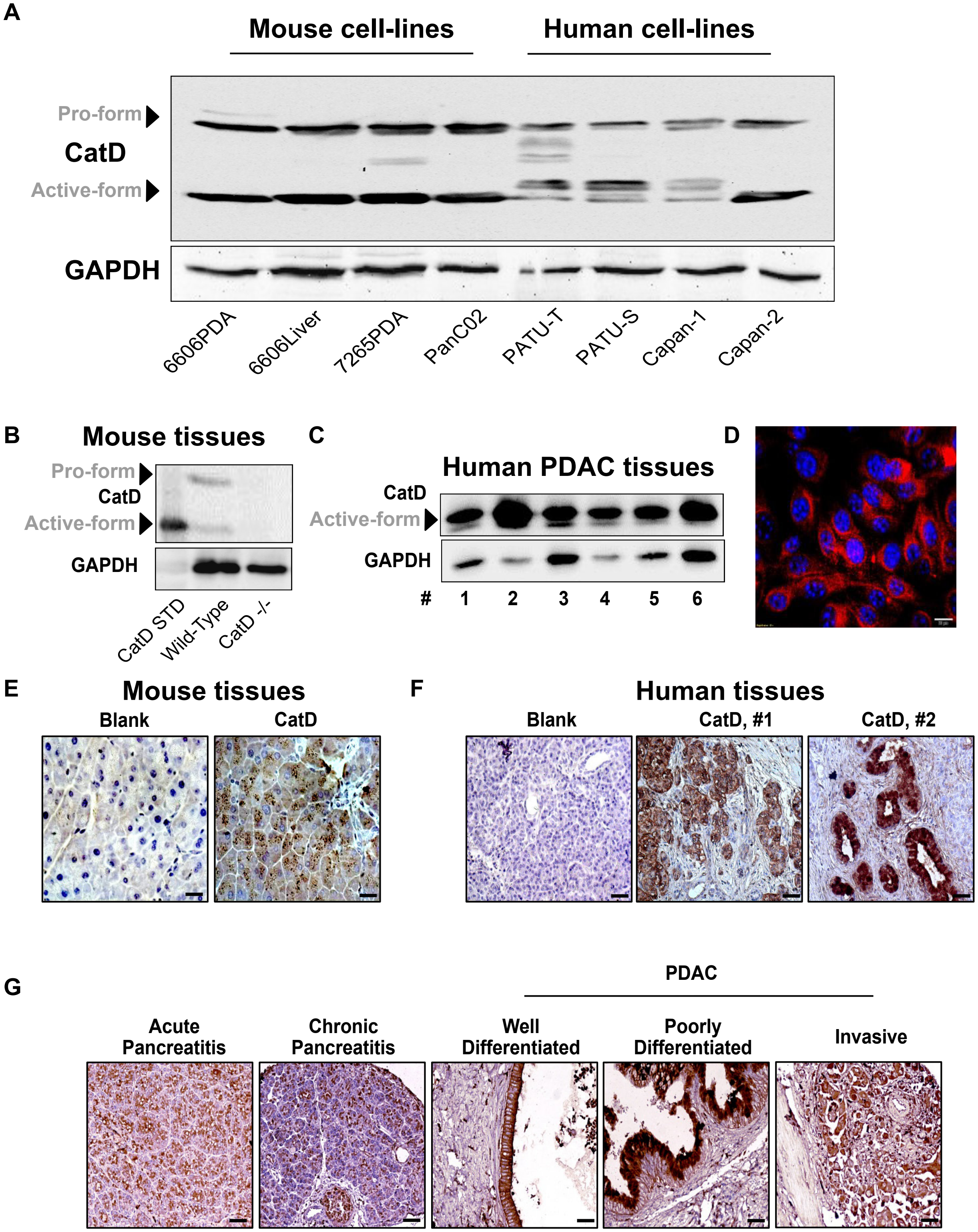

Supplementary figure 1: CatD antibody validation. (A) CatD expression in mouse and human cell-lines. **(B)** Specificity of CatD antibody when evaluated in CatD-/- mouse compared to WT mice. **(C)** Differential CatD expression in different human PDAC tissues. **(D)** CatD expression evaluated by immunofluorescence in PaTu-T cell-lines. **(E)** Immunohistrochemistry of CatD in normal mouse pancreatic tissue**. (F)** Immunohistochemical staining of CatD in human PDAC tissue specifically to the tumour cells. **(G)** Differential expression of CatD in different stages of pancreatic cancer development in human PDAC. Scale bar = 50µm

Supplementary Figure 2

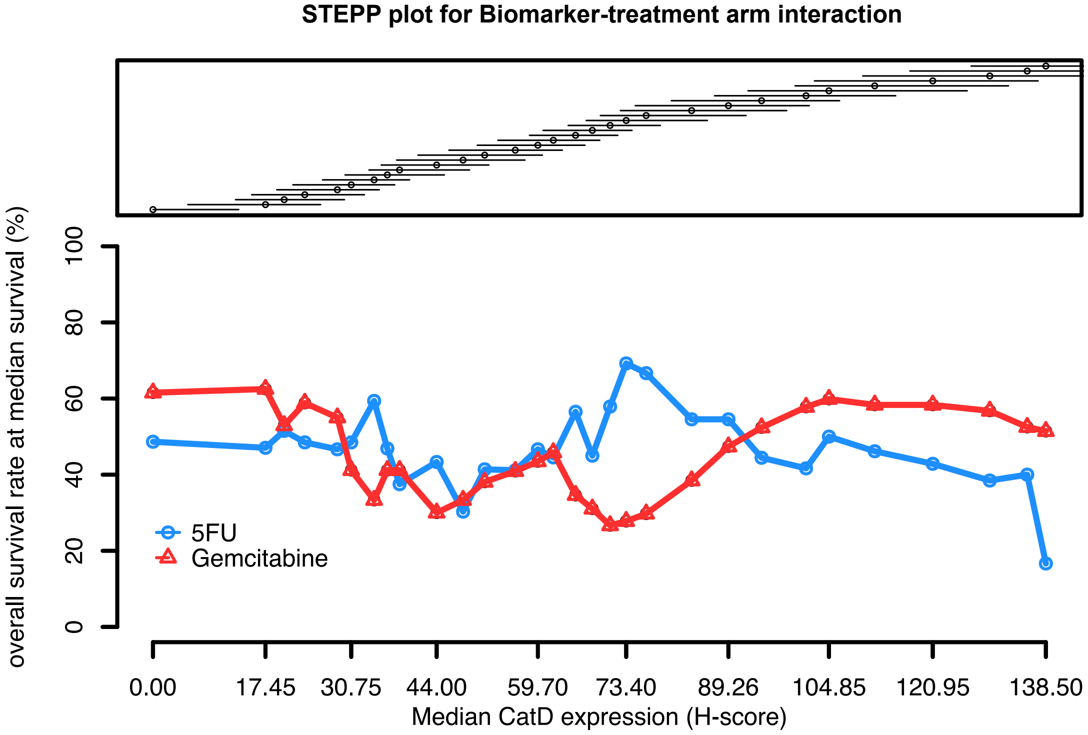

Supplemental figure 2: STEPP analysis to exclude a direct effect of treatment on CatD expression. To explore treatment-CatD expression heterogeneity, we performed sub-population treatment effect pattern plot (STEPP). To construct the overlapping subpopulations for the STEPP analysis, we set 50 patients as the size of each subpopulation with 40 as the number of patients included within consecutive overlapping subpopulations. We observed higher overall survival trend in patients with lower CatD values, especially in gemcitabine treated patients.

Supplementary Figure 3

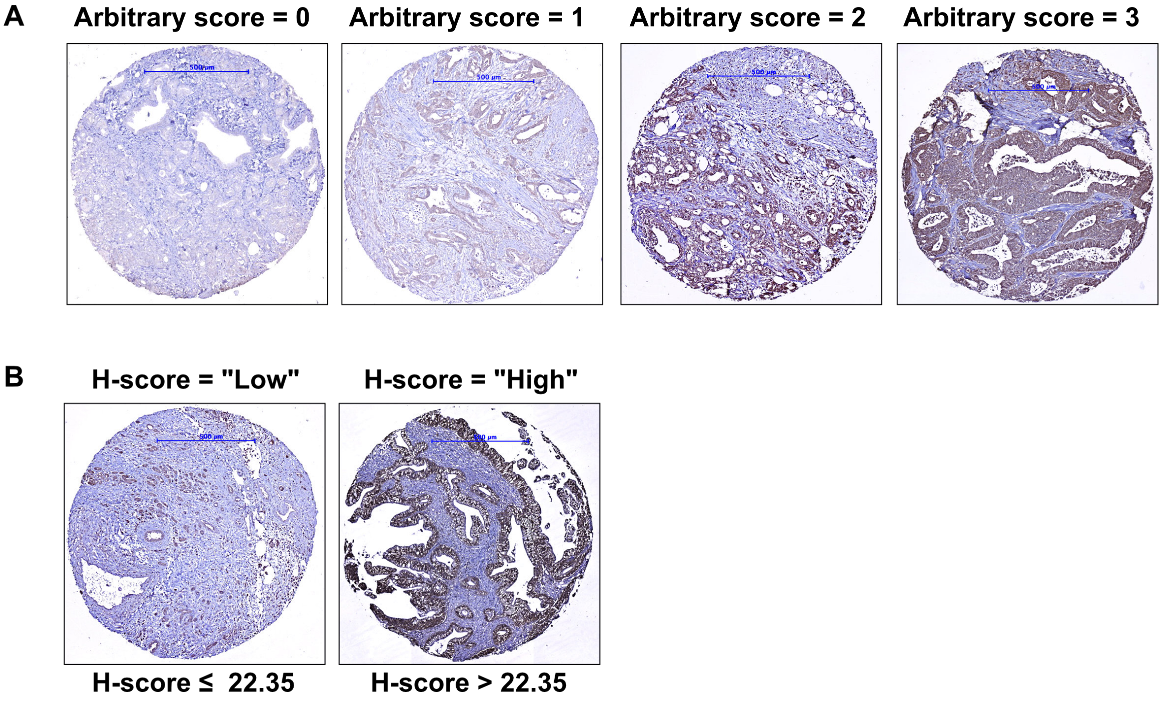

Supplementary figure 3: CatD staining in ESPAC-T patients' tissues. (A) Specimen images of CatD expression score in patient's cores. **(B)** Representative images of Low and High H-scored categorized patients.

Supplementary Figure 4

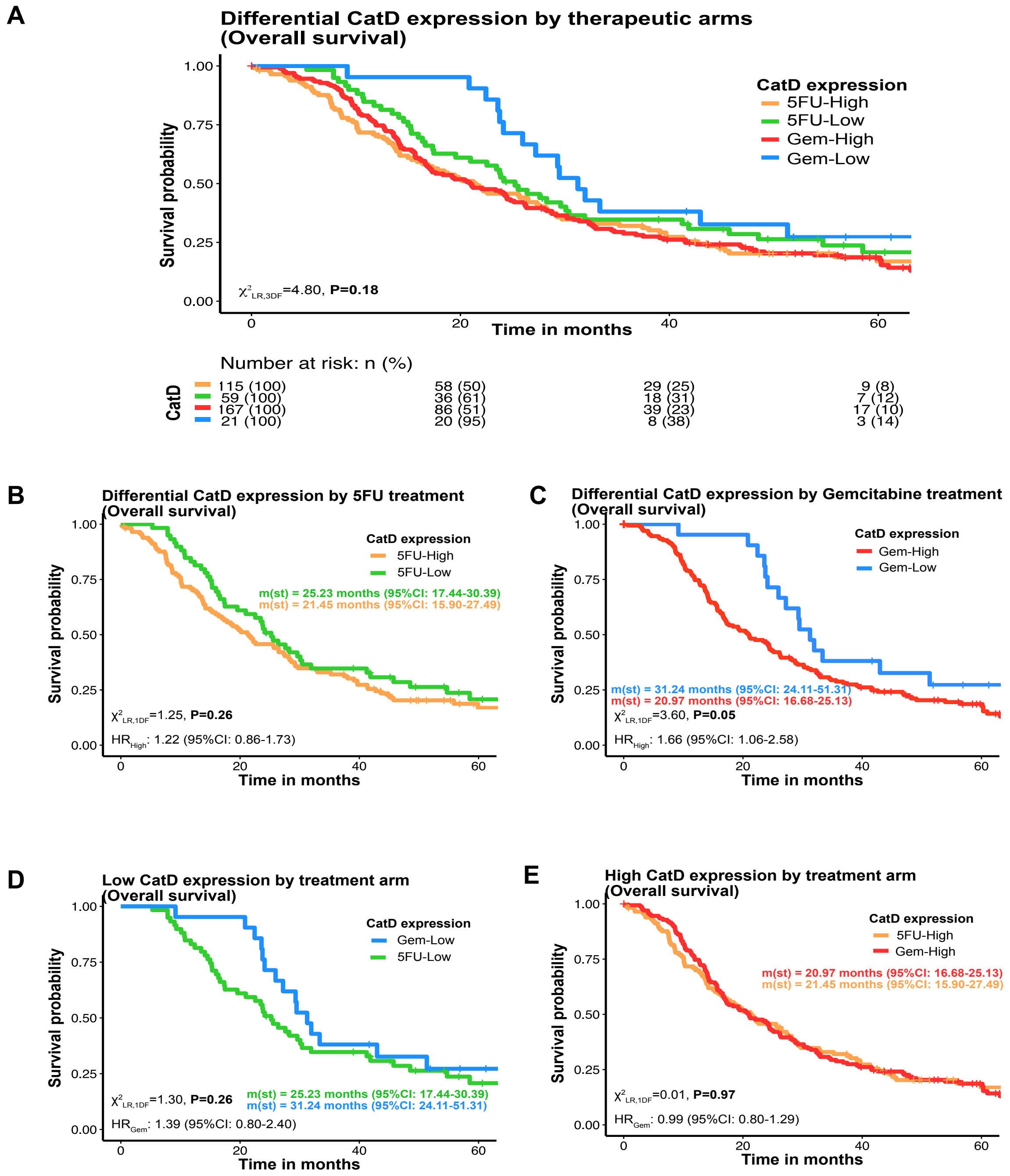

Supplementary figure 4: Stratification of differential CatD expression with treatment arms for overall survival. (A) Survival curves splits by treatment groups and CatD low/high expression for overall survival. Data is separated out for each of viewing for pairwise comparisons. **(B)** Differential CatD expression in 5FU/FA treatment arm. **(C)** Differential CatD

expression in Gemcitabine treatment arm. **(D)** Low CatD expression in different treatment arms. **(E)** High CatD expression in different treatment arms.

Supplementary Figure 5

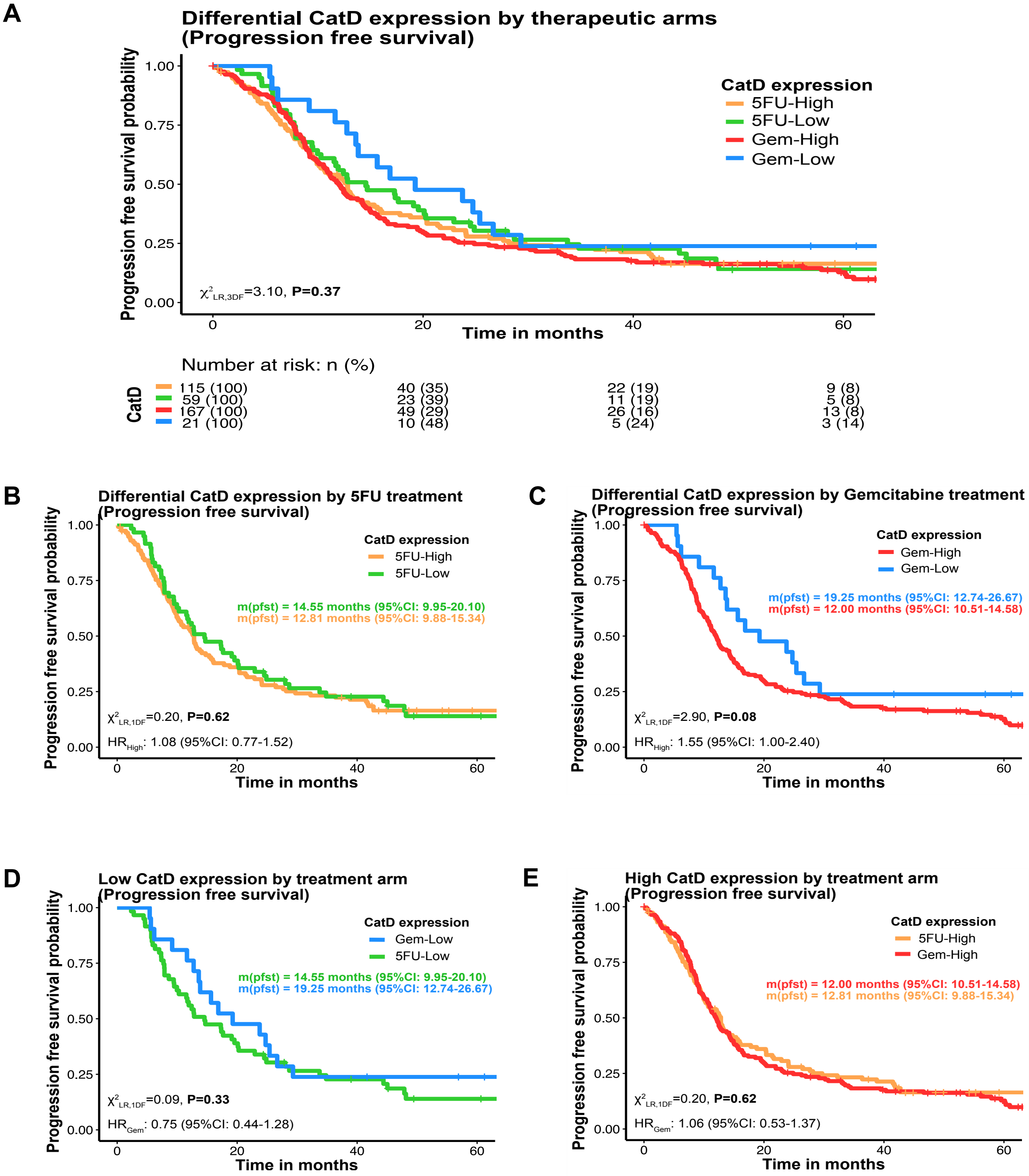

Supplementary figure 5: Stratification of differential CatD expression with treatment arms for progression free survival. (A) Progression free survival curves splits by treatment groups and CatD low/high expression for overall survival. Data is separated out for each of viewing for pairwise comparisons. **(B)** Differential CatD expression in 5FU/FA treatment arm. **(C)**

Differential CatD expression in Gemcitabine treatment arm. **(D)** Low CatD expression in different treatment arms. **(E)** High CatD expression in different treatment arms.

Supplementary Figure 6

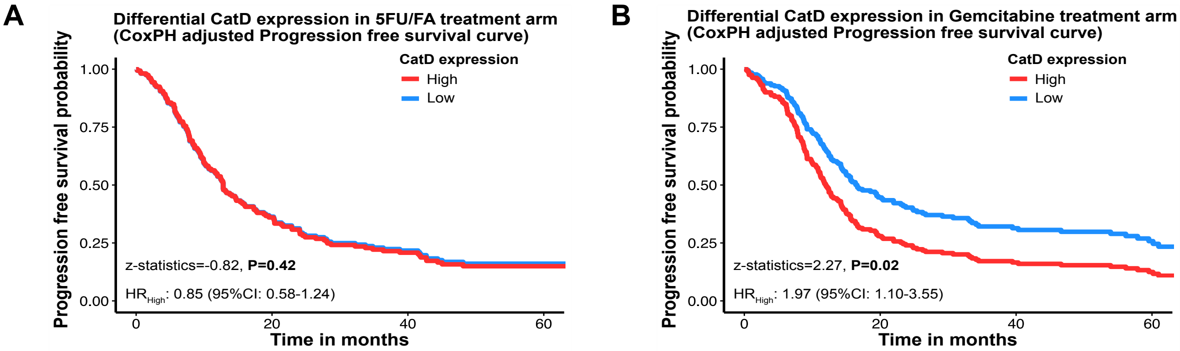

Supplementary figure 6: Multivariate Cox proportion hazard adjusted progression free survival differential CatD expression in ESPAC-Tplus cohort. (A) Multivariate cox proportional hazards regression analysis for event free survival stratified for 5FU/folinic acid treatment arm with CatD expression as one of the covariates along with resection margin, smoking status, lymph node invasion and local invasion as other independent covariates. **(B)** Multivariate cox proportional hazards regression analysis for progression free survival stratified for Gemcitabine treatment arm with CatD expression as one of the covariates along with resection margin, smoking status, lymph node invasion and local invasion as other independent covariates.

Supplementary Figure 7

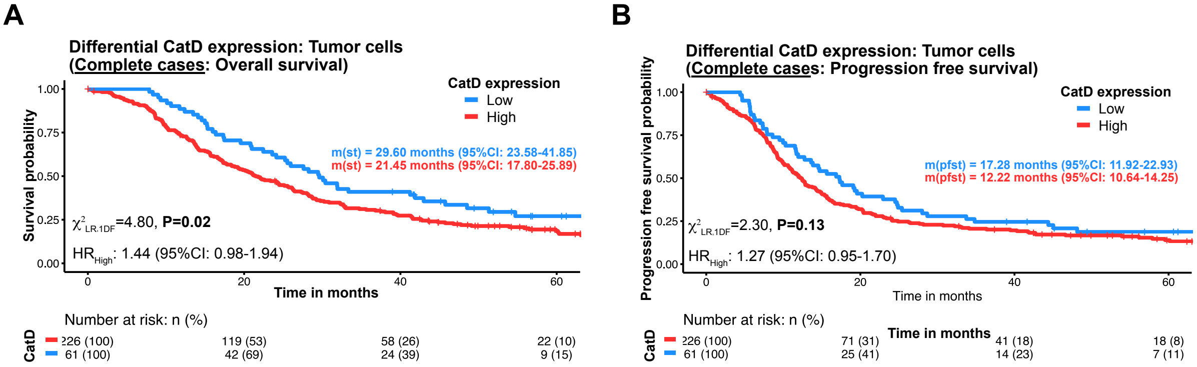

Supplementary figure 7: Association of CatD expression with overall survival in complete cases of ESPAC-Tplus cohort. (A) Overall survival curves split by differential CatD expression levels in the complete cases of ESPAC-Tplus cohort. All groups and the number of at-risk individuals is shown at the bottom of graph. **(B)** Progression free survival curves split by differential CatD expression levels in the complete cases of ESPAC-Tplus cohort. All groups and the number of at-risk individuals is shown at the bottom of graph. All statistical tests were log-rank analyses using two-sided χ^2 tests. CI = confidence level, HR: Hazard ratio.

Supplementary Figure 8

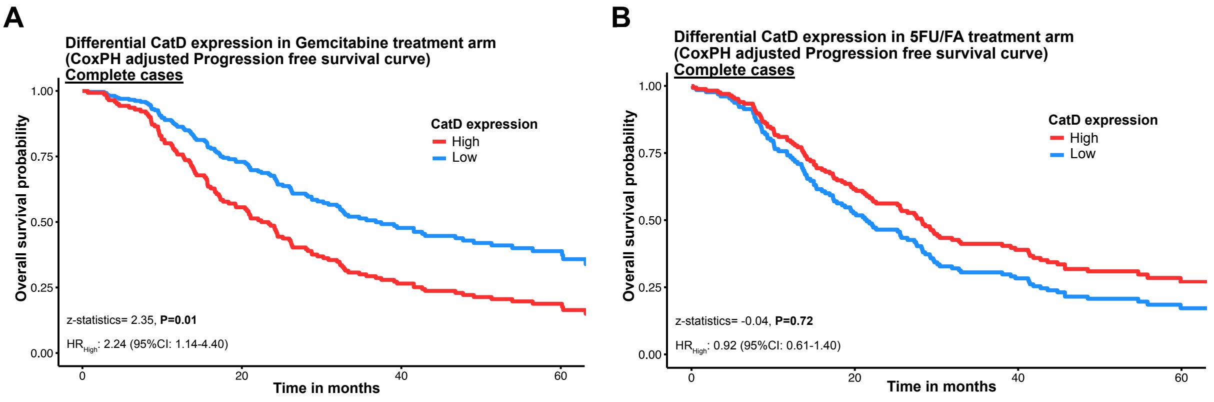

Supplementary figure 8: Multivariate Cox proportion hazard adjusted overall survival differential CatD expression in complete cases of ESPAC-Tplus cohort. (A) Multivariate cox proportional hazards regression analysis for overall survival stratified for gemcitabine treatment arm with CatD expression as one of the covariates along with resection margin, smoking status, lymph node invasion and local invasion as other independent covariates in complete cases of ESPAC-Tplus cohort. **(B)** Multivariate cox proportional hazards regression analysis for overall survival stratified for 5FU/FA treatment arm with CatD expression as one of the covariates along with resection margin, smoking status, lymph node invasion and local invasion as other independent covariates in complete cases of ESPAC-Tplus cohort. All statistical tests were log-rank analyses using two-sided χ^2 tests. CI = confidence level, HR: Hazard ratio.

Supplementary Figure 9

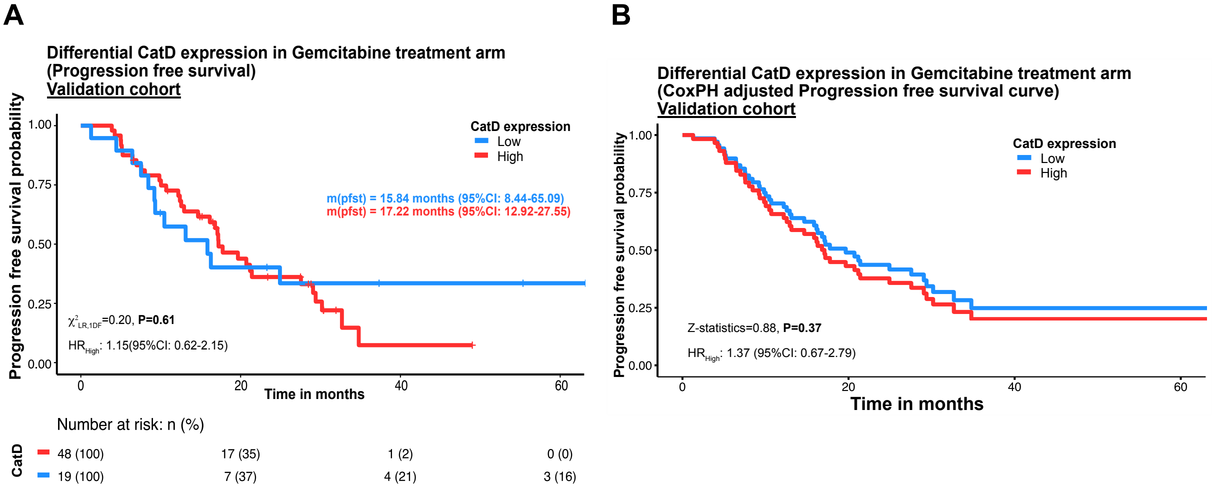

Supplementary figure 9: Association of CatD expression with progression free survival in resected PDAC patients in an independent validation cohort. (A) Progression free survival curves split by differential CatD expression levels in the validation cohort (univariate analysis). All groups and the number of at-risk individuals are shown at the bottom of graph. **(B)** Multivariate cox proportional hazards regression analysis for progression free survival stratified for gemcitabine treatment arm in validation cohort with CatD expression as one of the covariates along with sex, lymph node invasion and local invasion status as other independent covariates. All statistical tests were log-rank analyses using two-sided χ^2 tests. CI = confidence level, HR: Hazard ratio.

Supplementary Figure 10

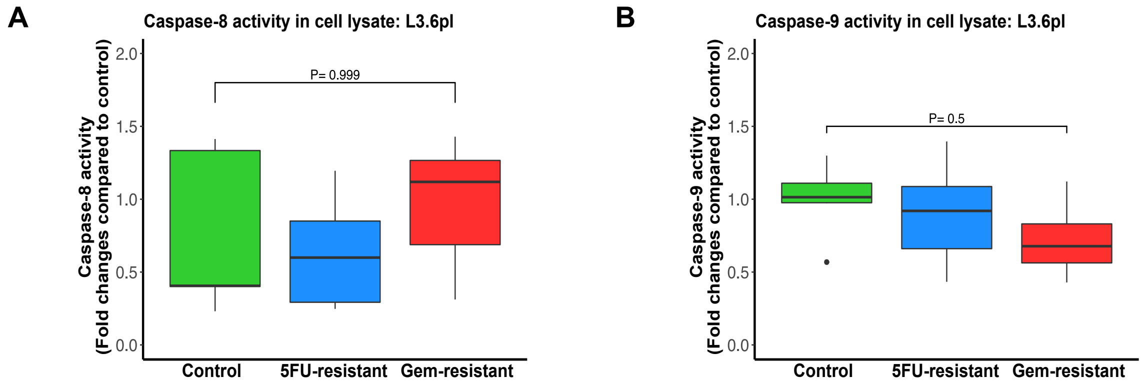

Supplementary figure 10: Influence of CatD expression and activity in gemcitabine resistance. Fluorogenic activity measurement of caspase 8 **(A)** and caspase 9 **(B)** in control, 5FU resistant and gemcitabine resistant cell-lines. Data represented as fold changes compared to control. *p<0.05, **p<0.01, ***p<0.005 considered significant Vs respective controls.
